# A novel framework for engineering protein loops exploring length and compositional variation

**DOI:** 10.1038/s41598-021-88708-4

**Published:** 2021-04-28

**Authors:** Pedro A. G. Tizei, Emma Harris, Shamal Withanage, Marleen Renders, Vitor B. Pinheiro

**Affiliations:** 1 Department of Structural and Molecular Biology, University College London, Gower Street, London, WC1E 6BT UK; 2 Department of Biological Sciences, University of London, Malet Street, Birkbeck, WC1E 7HX UK; 3 KU Leuven, Rega Institute for Medical Research, Medicinal Chemistry, Herestraat 49, Box 1041, 3000 Leuven, Belgium

**Keywords:** Molecular engineering, Protein design, Biological techniques, Biotechnology, Molecular biology

## Abstract

Insertions and deletions (indels) are known to affect function, biophysical properties and substrate specificity of enzymes, and they play a central role in evolution. Despite such clear significance, this class of mutation remains an underexploited tool in protein engineering with few available platforms capable of systematically generating and analysing libraries of varying sequence composition and length. We present a novel DNA assembly platform (InDel assembly), based on cycles of endonuclease restriction digestion and ligation of standardised dsDNA building blocks, that can generate libraries exploring both composition and sequence length variation. In addition, we developed a framework to analyse the output of selection from InDel-generated libraries, combining next generation sequencing and alignment-free strategies for sequence analysis. We demonstrate the approach by engineering the well-characterized TEM-1 β-lactamase Ω-loop, involved in substrate specificity, identifying multiple novel extended spectrum β-lactamases with loops of modified length and composition—areas of the sequence space not previously explored. Together, the InDel assembly and analysis platforms provide an efficient route to engineer protein loops or linkers where sequence length and composition are both essential functional parameters.

## Introduction

Directed evolution is a powerful tool for optimizing, altering or isolating novel function in proteins and nucleic acids^1, 2^. Cycles of sequence diversification to generate libraries followed by partitioning of those populations through selection, enable a desired function to be isolated and systematically optimised. Directed evolution is therefore a walk across sequence space with library quality and diversity being key factors in how efficiently that search can be carried out and in how much of the available sequence space can be explored.

Commonly used library synthesis methods focus on efficiently sampling sequence landscapes of fixed length, creating libraries that vary in composition (i.e. amino acid sequences on proteins) but not in length. Those methods vary in cost, in how that diversity is distributed and in the level of customization (i.e. redundancies, biases and coverage) that can be implemented^3, 4^. PCR-based methods using degenerate primers provide a cost-effective route towards creating focused (i.e. that target a small number of clustered sites) high-quality libraries^5, 6^ but can rapidly become laborious to synthesise, requiring large numbers of oligonucleotides to reach highly complex libraries where specific positional biases (e.g. Gly and Pro) or specific incorporation patterns (e.g. Ser only after Gly) can only be achieved by compromising library quality. For highly complex libraries, PCR-based methods cannot rival commercial high-throughput DNA synthesis platforms^7^, or DNA assembly methods that rely on the incorporation of individual triplets^8–10^ for customization. Methods that have been developed to exploit changes in length—be it through modifying oligo synthesis^11^, by using insertion and excision cycles of engineered transposons^12–16^ or by combining chemical and enzymatic approaches^17^—can deliver high quality libraries (i.e. where most indels do not affect the reading frame) but most need specialist equipment or are only able to generate a limited spectrum of indel mutations (summarised in Table 1).

**Table 1:** Principle and mutational scope of a selected range of methods focused on the generation of libraries that tolerate length and compositional variation.

Method	Principle and mutational scope
COBARDE^11^	Orthogonal protecting groups in oligonucleotide synthesis. One protecting group is used in the high efficiency synthesis of the oligonucleotide while a second one, introduced stochastically, allows synthesis to be interrupted. Libraries contain only deletions from a maximum length synthesis program. Deletions can vary in length and depend only on the frequency and concentration of stochastically used protecting group
Codon deletion mutagenesis (CDM)^13^	Engineered asymmetric Mini-Mu transposon random integration followed by Type IIS restriction endonuclease cleavage and target re-ligation. Transposon sequence (position of Type IIS recognition site) determines size of deletion, ranging from three to 15 nucleotides. In frame deletions can be selected ensuring only deletions are introduced in library
MuDel^16^	Mini-Mu transposon random integration followed by Type IIS restriction endonuclease cleavage and target re-ligation. Transposon excision removes three nucleotides, creating a codon-length deletion but not necessarily in frame. Mutations therefore include deletions as well as standard mutations in neighbouring residues
Pentapeptide scanning mutagenesis (PSM)^15^	Tn4430 transposon random insertion followed by orthodox Type II restriction endonuclease cleavage and target re-ligation. The use of orthodox endonucleases creates fixed “scar” sequences on the DNA level that limit the diversity of the inserted pentapeptide. Method is limited to 5-residue insertions
RAISE^18^	Terminal transferases (TdT) are used to introduce random sequences at the end of dsDNA fragments of the target DNA. Self-priming PCR is subsequently used to reassemble the target DNA incorporating TdT-generated material. Libraries introduce both sequence and length variation randomly in the library which can include both deletions and insertions
Random Elongation mutagenesis (REM)^19^	Short double-stranded DNA fragments containing sequence diversity are introduced via orthodox Type II restriction endonucleases to create terminal in frame insertions. The use of orthodox Type II restriction endonucleases introduces fixed “scar” sequences in generating a library. Selection of the dsDNA fragment offers control over the diversity and length of the inserted sequence
RID mutagenesis^17^	Circularized single-stranded target DNA is chemically cleaved to linearise them. Adaptor oligonucleotides (harbouring Type IIS restriction endonuclease sites) are ligated to the cleaved target DNA and PCR amplified. Circularization and removal of adaptor sequences result in a concomitant 3-base deletion (from the target) and an insertion (controlled by the sequence in the adaptors)
TRIAD^14^	The method expands on the MuDel approach introducing adaptors after the restriction endonuclease removal of transposon sequences which lead to larger deletions (up to 3 residues) or insertions (up to 3 residues). Mutations need not be in frame and adaptors define the sequence being inserted (and can include sequence variation)
TRINS^20^	Circularized single-stranded DNA fragments of the intended target are used as templates for limited rolling circle amplification. PCR assembly of fragments generates libraries of the target genes containing random tandem repeats of gene sequences. TRINS libraries reported show a large variation in insertion lengths, which need not be in frame
InDel Assembly (this study)	Additive synthesis of the target DNA using building blocks that are stochastically incorporated in the reaction. Mutations include deletions from a maximal length assembly but can introduce sequence variation by mixture of building blocks. While building blocks presented here are three nucleotide long, the length of the building blocks can be varied, enhancing the library complexity that can be obtained

In nature, insertions and deletions are less frequent than substitutions^21, 22^, which may partly reflect their greater impact on protein structure^23^ and function^24^. Nonetheless, it is widely accepted that indels have key roles in protein evolution particularly where they allow significant changes in protein function while minimising the number of mutational intermediates^24, 25^. Indels are also particularly relevant to the engineering of systems in which loops contribute significantly to protein function, such as the H3 loop in antibodies^26^ or the loops in TIM barrel enzymes^24^. In those circumstances, methods that target loop composition as well as length are essential for efficient functional optimization.

Traditional methods can address the problem by brute force, sampling sequence space through the use of multiple libraries of varying sequence composition, each with a given length^27^. Nonetheless, the challenge for analysing the output of selection of such library remains largely unaddressed. Indels introduce ambiguity in alignment since the exact position of a gap, in the absence of relevant phylogenetic or structural data^28^, cannot be inferred—and this limitation is highly relevant when aligning a large number of short sequences such as the output of selection when optimising a binding loop. Instead, selection can be carried out until population diversity is sufficiently low that analysis is redundant, or by analyzing single-length landscapes^29^.

The first approach increases the possibility of failure (e.g. parasites in selection), and can lead to the isolation of suboptimal variants because of experimental biases and inadequate sampling in the early selection rounds. Deep sequencing of the libraries captures the complexity of the available functional space in early rounds but, by analysing single-length landscapes individually, some information is inevitably lost, potentially by masking motifs present in multiple lengths or by increasing the possibility of false positives in sparsely sampled landscapes.

Here, we present a combination of (1) a cost-effective DNA assembly of high-quality, highly customizable focused libraries capable of sampling both length and compositional variation, and (2) a robust analytical framework that utilizes deep sequencing of pre- and post-selection libraries to identify enriched motifs across different length libraries. Together, they establish a powerful strategy to efficiently engineer loops and linkers from repertoires that vary in both length and composition.

## Results

### DNA assembly by cycles of restriction digestion and ligation: the InDel assembly

**Figure 1:**
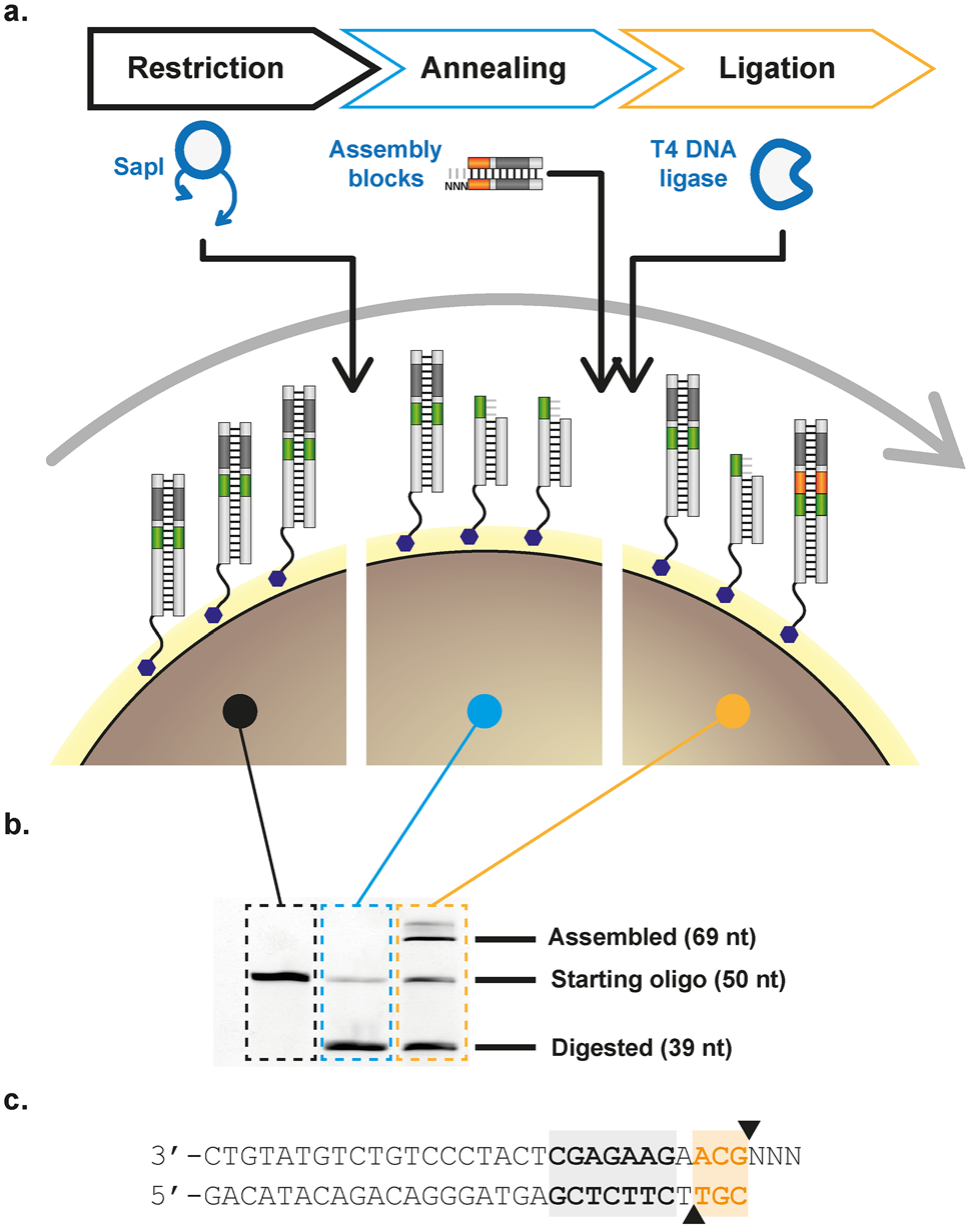
Producing length and compositional variation with InDel assembly. (**a**) At each assembly cycle, dsDNA templates bound to the paramagnetic beads are restricted with *Sap*I (a type IIs endonuclease), building blocks annealed and ligated. After ligation, the cycle can be restarted. Compositional variation is achieved primarily by combining pools of different building blocks. (**b**) Denaturing gel electrophoresis of fluorescently labelled template across the different steps of the cycle show that restriction digestion and ligation are not carried out to completion in any step, underpinning the length variation of the resulting libraries. (**c**) Sequence of a building block. A long double-stranded region is used to stabilize building block annealing and ensure efficient endonuclease activity. *Sap*I recognition site is shown in grey and restriction sites as black triangles. The overhang depicted would add GCA, coding for alanine to the growing chain—further information in Supplementary Table 3.

InDel assembly relies on cycles of DNA restriction digestion and ligation to progressively assemble a DNA library on paramagnetic beads, which serve as solid support and facilitate sample handling. The assembly starts with a biotinylated dsDNA template, encoding the starting point of the library and a recognition site for a type IIs endonuclease, bound to paramagnetic streptavidin-coated beads (Fig. 1a).

Type IIs restriction endonucleases bind non-palindromic recognition sequences and cleave dsDNA specifically, generating blunt or short single-stranded overhangs, which have been extensively exploited in molecular biology for ‘seamless’ cloning, as in Golden Gate assembly^30^. In InDel assembly, *Sap*I (a type IIs endonuclease) is used to digest the template, generating a 3-base ssDNA overhang and removing its recognition site from the bead-bound template.

The overhang generated by that cleavage enables the ligation of a standardized dsDNA building block with compatible overhangs. Building blocks (Fig. 1c) have been designed to have a degenerate overhang (minimising template sequence constraints) and a *Sap*I site, which enables the assembly cycle to be restarted.

Crucially, a triplet is placed between the overhang and the *Sap*I cleavage site, ensuring an increase in sequence length and maintaining the underlying reading frame. Variation of the triplet, which can be achieved by concomitantly adding two or more building blocks (in practice, any custom mixture thereof) to the ligation step, therefore leads to sequence variation in the resulting library. Like Sloning^10^ and ProxiMAX^9^, InDel is capable of delivering a highly flexible library since the building blocks can be mixed in any ratio and can incorporate any sequence and length of DNA—and hence could also be explored for protein fragment assembly.

Because no restriction digestion or ligation reaction is carried out to completion in the system (Fig. 1a,b), the library accumulates not only compositional but also length variation with a fraction of available templates not extended in each assembly cycle. The resulting InDel-assembled library therefore is more complex than what can be achieved by commercial platforms—it generates diversity comparable to COBARDE^11^ but requires no specialist equipment or reagents for library assembly.

Each reaction step in the InDel assembly was validated and optimised using fluorescently-labelled templates, with restriction digestion and ligation monitored by shifts in mobility of the fluorescent oligo in denaturing PAGE (Fig. 1b). We optimized ligation (Supplementary Fig. 1) and restriction digestion conditions, explored building block topology (i.e. hairpins or dsDNA from annealed strands), explored creating library degeneracy through concomitant addition of multiple blocks, and other reaction parameters.

Optimised reactions suggested between 20 and 50% assembly efficiency per cycle could be obtained, based on the densitometric analysis of PAGE assays (e.g. Fig. 1b and Supplementary Fig. 1b). However later deep sequence analysis of synthesized libraries determined that the incorporation efficiency per cycle was lower (Supplementary Fig. 2a)—probably the result of extended sample handling and the limited activity of *Sap*I in extended reactions. Further optimization of assembly conditions, in the form of the method presented here, yielded assembly efficiencies close to 20% per cycle (Supplementary Fig. 2d). Codon biases were observed but varied between assembled libraries, suggesting that it is not a limiting factor in assembly and, as with similar platforms^9^, can be further optimised if needed.

### TEM-1 Ω-loop functional sequence space includes loops of different length as well as different composition

**Figure 2:**
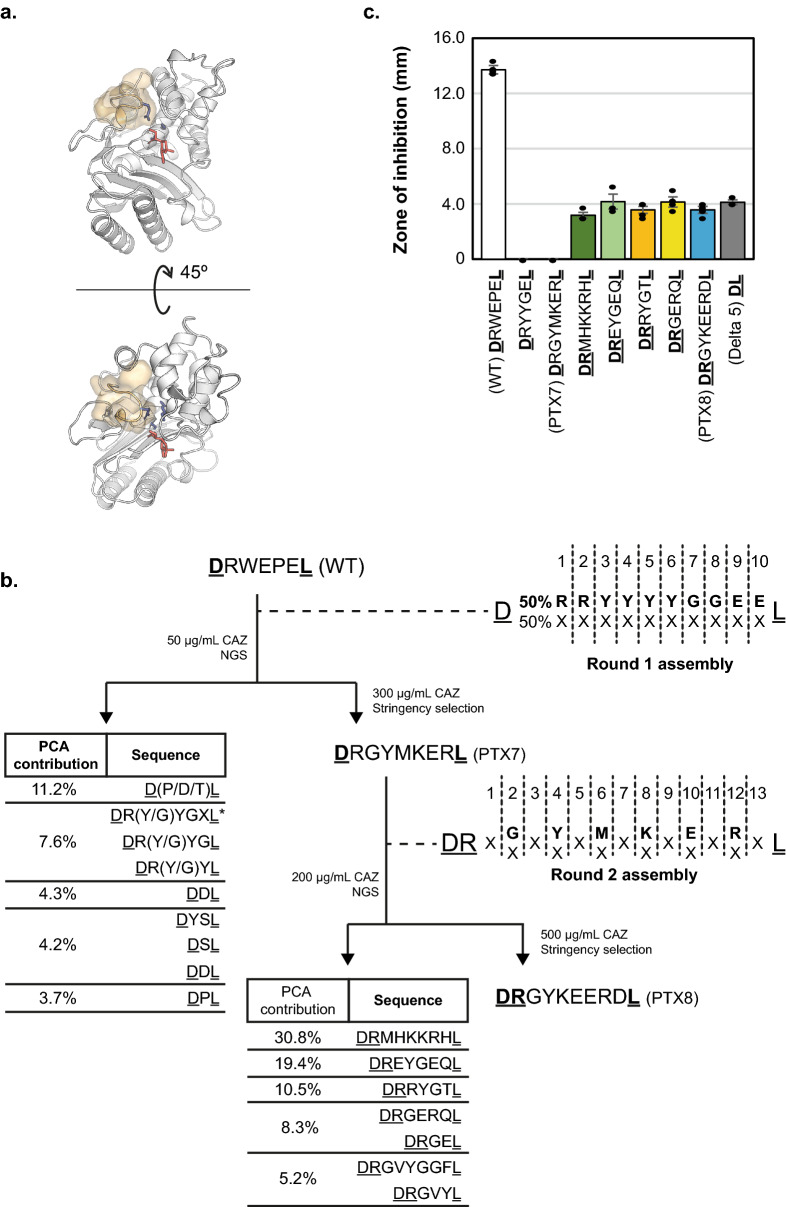
Directed evolution of TEM-1 Ω-loop variants with altered substrate specificity. (**a**) TEM-1 beta-lactamase in complex to substrate analogue inhibitor (PDB: 1TEM) as generated by PyMOL^39^. Inhibitor is shown in the TEM-1 active site in black, and side chains of key catalytic residues (S70, K73 and E166) are shown. The Ω-loop is shown in grey and superimposed to its translucent space-filling representation. (**b**) Summary of the directed evolution of TEM-1. A 1st round library, biased towards the generation of RYYGE variant was assembled (each cycle containing 50% of a specific building block and 50% of a mixture of the remaining 19 blocks) and selected. Next generation sequencing (NGS) confirms RYYGE was significantly enriched (DRYYGEL was the 16th most frequent sequence post-selection and picked up in the 2nd PCA dimension—see Supplementary Table 1). PTX7, isolated from a high-stringency selection, was used as seed for the 2nd round library. Assembly of the second-round library interspersed cycles of random insertion (all possible blocks in equimolar concentrations) and the same 50%/50% approach used in round 1. PTX8 and sequences from the top four PCA (Principal component analysis) dimensions were further characterized. (**c**) Ceftazidime resistance of selected variants, measured by inhibition of growth around antibiotic-soaked paper discs—higher values indicate lower resistance (n = 3, error bars represent S.E.M). Underlined residues represent the invariant edges of the assembly, constant in each library and required for library amplification.

Having established the assembly platform, we chose the β-lactamase TEM-1 to demonstrate its potential. TEM-1 is a well-characterized enzyme^31, 32^ that due to its ease of selection, wide range of available substrate analogues and its clinical relevance, has long been used as a model in directed evolution^33, 34^. In particular, TEM-1 contains a short flexible loop which is part of its active site (the Ω-loop, _164_RWEPE_168_—Fig. 2a) and has been implicated in substrate specificity. To date, engineering of the Ω-loop has focused mostly on exploring variation in composition of the loop, culminating on the successful isolation of _164_RYYGE_168_, a variant highly-resistant to the cephem ceftazidime^35, 36^. Deletions^35^, long insertions (> 5 residues)^37^ and circular permutation^38^ at the Ω-loop have also been shown to produce cephem-resistant variants, albeit at substantially lower levels than _164_RYYGE_168._

Our initial goal was to explore the sequence neighbourhood of the previously reported _164_RYYGE_168_ variant with a view towards demonstrating assembly and selection, that is generating a library enriched for sequences harbouring one or two mutations away from the RYYGE motif. Based on our early estimates of 50% assembly efficiency per cycle (Fig. 1b), we assembled a 10-cycle InDel library using biased mixes of building blocks—50% coding for the desired target residue and the remaining 50% divided between the remaining 19 coding triplets (Fig. 2b). Based on a simple binomial model (described in Supplementary Note 1), the library was expected to fully sample all variants of up to four inserted codons and sample longer landscapes increasingly more sparsely—but always biased towards sequences related to _164_RYYGE_168_ (Supplementary Fig. 2).

Assembled libraries were cloned into a TEM-1 backbone harbouring the M182T stabilizing mutation^34^. Selection was carried out by plating cells transformed with the TEM-1 library directly on media supplemented with 50 μg/mL ceftazidime—below the minimal inhibitory concentration for the _164_RYYGE_168_ variant harbouring the stabilizing mutation (Supplementary Fig. 4).

Sequencing of pre- and post-selection library confirmed that the _164_RYYGE_168_ variant was present in the starting library, albeit at a frequency lower than expected (5 reads in 2.3 million—0.0002% of the population), and was significantly enriched on selection (314 reads in 230,000—0.14%)—an enrichment score of 8460 (in the 99th percentile of a distribution of enrichment Z-scores based on the comparison of two Poisson distributions). Enrichment of the TEM-1 _164_RYYGE_168_ variant in selection clearly demonstrates that InDel assembly and selection can recapitulate previous engineering efforts at altering the substrate specificity of TEM-1.

In parallel with deep sequencing of the libraries, we also increased the stringency of selection by plating the library at higher ceftazidime concentrations. At ceftazidime concentrations of 300 μg/mL, a single variant was isolated: _164_RGYMKER_168b_ (adopting antibody annotation to describe insertions^40^—see materials and methods for more details), differing in both composition and length from wild-type (_164_RWEPE_168_) and engineered TEM-1 (_164_RYYGE_168_) sequences. Undetected in the input library, _164_RGYMKER_168b_ represented approximately 0.004% of the selected library (9 reads in 230,000) and displayed a resistance profile comparable to that of the previously engineered _164_RYYGE_168_ (Fig. 2c). Isolation of _164_RGYMKER_168b_ TEM-1 variant confirms that high levels of ceftazidime resistance are not unique to _164_RYYGE_168_ and further validate that loop length is a crucial parameter in protein engineering.

### InDel-assembled libraries are high quality and efficiently sample sequence landscapes of different lengths

In addition to enabling us to look at the impact of selection, deep sequencing of pre- and post-selection InDel-assembled libraries allowed the quality of the libraries to be assessed, including biases, coverage and assembly errors (e.g. frameshifts).

**Figure 3:**
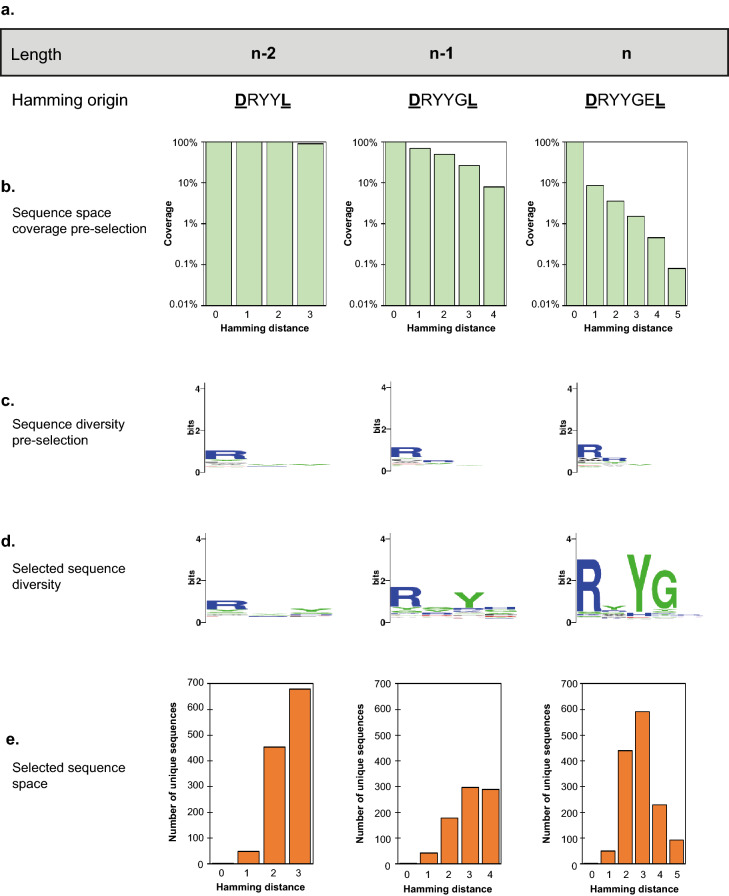
InDel assembly coverage of sequence space neighbouring RYYGE and impact of selection. (**a**) The available sequence space is split into fixed-length landscapes and each analysed separately using the most frequent variant of the desired length as the origin for Hamming distances—a simple count of the number of mutations away from the reference sequence. (**b**) The biased synthesis used in the InDel assembly of this first library ensured the sequence neighbourhood of the target RYYGE sequence was efficiently explored with lower Hamming distances more effectively sampled. (**c**) The library remains diverse with only minimal bias for arginine incorporation in the first position, as predicted. The height of each residue in the logo is a measure of their frequency at that position. (**d**) Selection clearly enriches for an RXY motif particularly in *n-1* and *n* landscapes. (**e**) Hamming distances to other unique sequences obtained in each landscape after selection, highlighting the presence of a local peak around RYYGE.

The pre-selection libraries had the expected biases introduced in assembly (Fig. 2b and Supplementary Fig. 2), with preferred codons being overrepresented in the library—e.g. R and Y in the round 1 library (Supplementary Fig. 4). Pre-selection sequence diversity was high, with only the first R incorporation showing significant conservation (Fig. 3c), and showed complete or heavily biased coverage towards the target _164_RYYGE_168_ motif (Fig. 3b).

Post-selection, consensus motifs can be determined for each single-length sequence landscape but are strongest in the 5-mer landscape as RXYGX (matching to the previously described RYYGE motif), seen both as an increase in information content (Fig. 3d) and in the distribution of selected sequences across the landscape (Fig. 3e). This further confirms _164_RYYGE_168_ as a functional ‘peak’ in the 5-mer landscape.

Analysis of enrichment also suggests that the functional space of the TEM-1 Ω-loop is densely populated, with multiple functional motifs present in different loop lengths—and not necessarily related to the wild-type _164_RWEPE_168_ or engineered _164_RYYGE_168_ motifs (Figs. 2c, 3 and Supplementary Fig. 3). This is further supported by our isolation of the _164_RGYMKER_168b_ variant, which differs from all previously reported variants in both composition and length, highlighting the power of InDel to navigate the sequence space in which length is an additional design parameter.

### Alignment-free sequence analysis improves identification of enriched motifs

While the use of deep sequencing to map functional landscapes^41^ and to accelerate directed evolution^42^ is well-established, current methods do not perform well for short libraries that vary in both length and composition^43^. Stratifying the library into fixed-length repertoires for analysis^29^ or using indels to contribute to a mismatch score (i.e. Hamming distance) have been applied to the analysis of length and compositional variation. However, length variation in directed evolution is generally discarded^44^ because of difficulties in positioning gaps in the resulting alignments^43^.

We therefore set out to develop an alignment-free sequence analysis method based on subdivision of sequence strings into short reading windows (*k*-mers) to extract information from sequencing data spanning multiple fixed-length sequence landscapes. *K*-mer based methods are integral components of large sequence comparison methods^45^ as well as next generation sequence assembly^46^, allowing comparison of sequences of different lengths as well as reconstruction of sequence motifs—the two steps required to identify functional variants from the available InDel assembly libraries.

**Figure 4:**
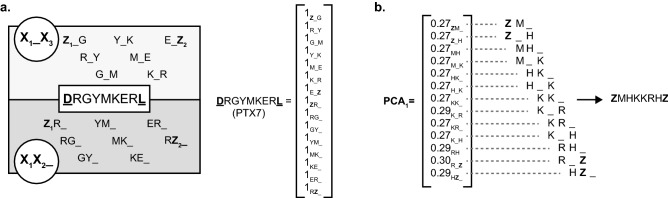
*K*-mer sequence decomposition and reconstruction. (**a**) Sequences can be decomposed into all possible masked 3-mers (i.e. X_1_X_2_X_3_ separated into X_1_X_2__ and X_1__X_3_) as shown for PTX7. Each masked 3-mer is counted generating an 882-dimension vector (non-zero elements shown). Vectors are normalized (divided by $\sqrt {15}$ in the case of PTX7) and multiplied by their enrichment score. Principal component analysis (PCA) identifies enriched *k*-mers, which allow a sequence to be reconstructed. An arbitrary cut-off (0.1) can be used to minimize noise and facilitate assembly. (**b**) The sequence reconstruction of the first PCA component calculated from the second library selection.

We opted for using masked 3-mers (i.e. representing a 3-mer X_1_X_2_X_3_ as X_1_X_2__ and X_1__X_3_ by masking either the 3rd or 2nd residues)^47^ to analyze sequences, reducing computational burden (the possible 8000 3-mers are reduced to 800 possible masked 3-mers) without significant loss of information relevant for motif reconstruction. Based on analysis of ‘toy’ data sets (not shown), we chose to explicitly represent residues flanking the synthesized libraries (as Z_1_ and Z_2_—see Fig. 4a) in analysis, adding a further 82 possible *k*-mers but significantly improving the robustness of downstream motif reassembly.

Pre- and post-selection libraries were combined and each individual sequence, described by its masked 3-mer count, was treated as a column vector of 882 dimensions—the number of masked 3-mers used to describe sequence and library boundaries (Fig. 4a). Vectors were normalized and scaled by their Z-score as a measure of enrichment and as a proxy for function. Together, the entire output of selection is mapped onto a complex 882-dimensional space.

Principal component analysis (PCA) enabled us to deconvolute this complex space to identify, which combinations of the 882 dimensions (i.e. masked 3-mers) contribute the most to the distribution of the sequences within that space—in practice, allowing functional motifs to be reconstructed along individual PCA dimensions (Fig. 4b).

Highly enriched sequences contribute significantly to library variation and are identified in the first PCA dimensions (which account for the biggest variation in the data). Crucially, functional sequences that are related (i.e. share masked 3-mers) but not necessarily of the same length, cluster in this 882-dimensional space and are more readily picked up by analysis—Table 2 and Supplementary Table 1.

**Table 2:** Comparison of PCA-derived enriched sequences and NGS read frequency for the 2nd round library.

PCA dimension	PCA-derived motif	Most frequent match	Frequency ranking
1	ZMHKKRHZ	ZMHKKRHZ	1
2	ZEYGEQZ	ZEYGEQZ	2
3	ZRYGTZ	ZRYGTZ	3
4	ZGERQZ	ZGERQZ	4
	ZGEZ		
5	ZGVYGGFZ	ZGVYGGFZ	7
	ZGVYZ	ZGVYZ	8
6	ZAKERHZ	ZAKERHZ	5
	GVY	ZGVYZ	8
	ZE(V/K/R)XZ	ZEKERHZ	108
7	ZAKEXH	ZAKERHZ	5
	Z(A/G)YVZ	ZGYVZ	6
8	ZEEVHZ	ZEEVHZ	9
9	Z(A/W)(E/Y)EHRZ	ZWEGRQZ	10
	ZWEGR(Q)Z	ZAYEHRZ	12
	VEGRQ		
10	VXZ		
	Z(A/G)Y(Y/E)HRZ	ZGVYZ	8
	ZGY(Y)Z	ZAYEHRZ	12
	ZGAYEHRZ		

Motifs were reconstructed for the first 10 PCA dimensions and used to search the NGS results for the ranking of the highest enriched sequences (highest Z-scores). Because the second round post-selection library was significantly smaller and heavily biased, correlation between PCA and NGS frequency is very good. Some sequence variation and small motifs can still be seen.

Therefore, our approach identifies not only functional peaks that are restricted to a single length but also peaks that span different lengths, while considering contribution of submotifs (in longer loops) and degeneracies (i.e. non-conserved positions in a motif) that may also be contributing to selection. Motif reconstruction can be automated, with resulting sequences being tested for function or used as starting points for new libraries.

### The sequence neighbourhood of TEM-1 Ω-loop is densely populated with functional variants

Sequence analysis of the first round of selection identified a wide range of sequence motifs that were enriched in selection (Supplementary Table 1), suggesting that the sequence neighbourhood of the Ω-loop was more densely populated than previously expected. The short motifs identified however could be a reflection of the lower assembly efficiency of the first round (biasing the Ω-loop library towards short motifs) as well as the low stringency of selection used (enabling even moderately active variants to be selected).

We therefore decided to pursue a second round of selection to investigate the sequence space in the neighbourhood of _164_RGYMKER_168b_, including deletions, substitutions and insertions. Exploring that sequence space could easily be achieved with InDel by assembling a library alternating between fully degenerate (i.e. equal distribution of all available triplets) with biased (i.e. 50% of desired _164_RGYMKER_168b_ triplet and 50% of the remaining 19 triplets) cycles (Fig. 2b, Supplementary Fig. 5).

As before, selection was carried out by plating the assembled library onto solid media containing ceftazidime. Higher antibiotic concentrations were used (200 μg/mL) to increase selection stringency and 79 colonies were isolated. Pre- and post-selection libraries were sequenced and analysed with our *k*-mer approach (Table 2).

The top four candidates from the second-round library (DRMHKKRHL, DREYGEQL, DRRYGTL, DRGERQL), harbouring five to seven amino acids in the diversified region of the Ω-loop, were further characterized (Fig. 2c and Supplementary Fig. 5). All four variants (as well as a shortened loop variant Δ5) are significantly more resistant to ceftazidime than the wild-type enzyme.

Characterised variants show little sequence similarity to wild-type or engineered variants (_164_RYYGE_168_ and _164_RGYMKER_168b_). This diversity confirms that the sequence neighbourhood of the Ω-loop is densely populated with functional variants in multiple landscapes. It also demonstrates the potential of the InDel framework to efficiently explore sequence space varying both length and composition.

## Discussion

Length is, as yet, a poorly explored parameter in protein engineering despite clear evidence that loop and linker lengths are highly relevant to function on multiple systems. This is an acknowledged gap in the field^48^ and one that can only be adequately addressed by combining the synthesis of length-variable libraries with alignment-independent analysis tools. It is this combination that makes the InDel platform presented here a powerful new addition to the protein engineer’s toolset.

Generation of libraries containing length and compositional diversity is challenging. Cost-effective library generation methods have up to now compromised on library quality, either by the frequent introduction of frameshifts^20^, by their inability to target diversity to a specific region of the sequence^16, 19, 20^, or by their limited ability to customise library composition^20^ or length. High quality libraries (particularly when biased towards a given area of sequence space) invariably required specialist equipment and knowhow^16^, or the high cost of commercial synthesis to explicitly generate each length to be considered. InDel assembly bridges that gap, generating high quality libraries (that explore variation in both composition and length) in a low-cost protocol based on standard molecular biology tools.

At the other end of the engineering process, the benefits that deep sequencing can bring to protein engineering are limited in datasets that vary in both composition and length. Most commonly, motif detection is carried out by extracting the most highly-enriched sequences from the dataset^49^, where there is a correlation between enrichment and function in a working selection platform. This approach oversimplifies the functional landscape and is of limited use in subsequent designs. Because of the known issues around gap assignment in multiple sequence alignments^43^, the only other viable approach to analysing datasets that vary in length has been to partition them by length. The result is a series of length-specific analyses^29^, that again present an incomplete assessment of the functional space but can guide further development. The k-mer strategy developed as part of the InDel framework, bypasses those limitations identifying enriched motifs across different lengths, including non-contiguous motifs.

Applied to the beta-lactamase Ω-loop, we show that the combination of InDel assembly and *k*-mer-based analysis provide a powerful framework for navigating sequence space that is not otherwise accessible. Our results also provide further evidence that loops are highly evolvable^48^ and highlight how directed evolution of protein loops must take into account sequence spaces that straddle more than a single-length landscape. Effectively, InDel assembly, selection and *k-*mer analysis respectively provide ‘build’, ‘test’ and ‘learn’ steps of the Synthetic Biology cycle^50^ and could be automated to accelerate engineering of any protein function.

Although we present here an example of InDel assembly with triplets, which is ideal for generating amino-acid-steps in libraries of protein coding genes, the platform is compatible with building blocks of mixed length. That enables a vast host of combinatorial possibilities that could be applied to the directed evolution of nucleic acid aptamers, gene expression regulatory elements and fragment-based protein engineering.

## Materials and methods

### Assembly

All oligos used in InDel assembly were commercially synthesized (Integrated DNA Technologies). Assembly block oligos providing the 5′-end for ligation with the dsDNA template were phosphorylated in 100 µL reactions (1 nmol oligo per reaction) containing 1× NEB T4 DNA ligase reaction buffer and 1 µL NEB T4 polynucleotide kinase. Reactions were carried out for 3 h at 37 °C, followed by inactivation at 80 °C for 20 min. Oligos were phenol–chloroform extracted, ethanol precipitated, resuspended in 90 µL annealing buffer (10 mM Tris–HCl pH 8.0, 20 mM NaCl, 1 mM MgCl_2_, 0.01% Tween20) and annealed to 1 nmol of the complementary assembly block strand. Building blocks coding for different amino acids were mixed post annealing to create the desired incorporation proportions.

In parallel, 60 µL of MyOne C1 streptavidin-coated paramagnetic beads (Thermo Fisher Scientific) were washed twice in BWBS (5 mM Tris–HCl pH7.5, 0.5 mM EDTA, 1 M NaCl, 0.05% Tween20) and incubated at room temperature (in BWBS) for 30 min in a rotating incubator, to reduce background binding. After washing, 10 pmol of biotinylated dsDNA template oligos were added to the beads and incubated overnight at room temperature in a rotating incubator. Beads were washed in BWBS and transferred to a 0.5 mL microcentrifuge tube for assembly.

Bead-bound templates were digested with *Sap*I (NEB) in 100 µL reactions (10 µL 10× CutSmart buffer, 2 µL *Sap*I, 1 µL 1% Tween20) for 2 h at 37 °C with vortexing every 15–20 min to keep beads in suspension. Beads were isolated and washed once in BWBS. The supernatant containing *Sap*I, was retained and stored at 4 °C for subsequent assembly cycles.

The desired mixture of building blocks was added to the washed beads, incubated at 37 °C for 30 s, followed by an additional 30 s incubation at 4 °C. Supernatant containing the building blocks was removed and beads transferred to a ligation reaction. Ligations were carried out in 100 µL reactions (10 µL T4 DNA Ligase buffer, 12 µL 1,2-propanediol, 10 µL 30% PEG-8000, 1 µL T4 DNA Ligase, 1 µL 1% Tween20, 65 µL ddH_2_O) at 25 °C for 1 h, with vortexing every 15–20 min.

Beads were isolated, washed in BWBS and could then be taken to start a new assembly cycle. The supernatant containing the ligase reaction mixture was retained and stored at 4 °C for subsequent cycles.

The final assembly cycle used a modified dsDNA assembly block (a 3′ cap block) containing a priming site used for post-assembly library amplification. After ligation of the capping oligo, beads were resuspended in 50 µL BWBS for PCR amplification.

### Denaturing polyacrylamide gel electrophoresis

Assembly reactions carried out with FAM-labelled templates could be visualized after separation by denaturing PAGE. Gels were 15% acrylamide (19:1 acrylamide:bis-acrylamide) with 8 M urea in 1× TBE. An equal volume of loading buffer (98% formamide, 10 mM EDTA, 0.02% Orange G) was added to FAM-labelled templates, and sampled were incubated at 95 °C for 5 min before being loaded onto the gel. Gels were run at a constant current of 30 mA for 1.5–2 h. FAM-labelled oligos were detected by imaging on a Typhoon FLA 9500 scanner (GE Life Sciences). Images (Fig. 1b, SI Fig. S1) were analysed and prepared for publication using Fiji^51^. Levels were adjusted (whole figure and linear transformations only) prior to figure preparation, which was done using Adobe Illustrator.

### Library amplification and cloning

Assembled libraries were PCR amplified from beads in 50 µL reactions using 10 U MyTaq HS polymerase (Bioline), 0.2 µM each of oligos TEM1-InDel-AmpF(1/2, for corresponding rounds of selection) and TEM1-InDel-AmpR, 1 µL resuspended bead slurry from the assembled library, 1X MyTaq reaction buffer, and 1× CES enhancer solution^31^. Library amplifications were carried out with a 1 min denaturation at 95 °C, followed by 20 cycles of 15 s at 95 °C, 15 s at 55 °C, 30 s at 72 °C, ending with a 2 min final extension at 72 °C. PCR cycles were limited to 20 in library amplifications to minimize amplification biases and reduce likelihood of secondary mutations. Multiple reactions were carried out in parallel to ensure sufficient material for cloning could be generated and the oligos harbored BsaI overhangs for seamless DNA assembly.

Vector backbones were generated by iPCR in 50 µL reactions using 1 U Q5 Hot Start DNA Polymerase (NEB), 0.2 µM each of oligos Vec-TEM1-InDel-F and Vec-TEM1-InDel-R(1/2, for corresponding rounds of selection), 1 ng pTEM1-Cam vector template, 200 µM dNTPs, 1× Q5 reaction buffer, and 1× CES enhancer solution^31^. Vector amplifications were carried out with a 30 s denaturation at 98 °C, followed by 30 cycles of 10 s at 98 °C, 20 s at 68 °C, and 1.5 min at 72 °C, ending with a 2 min final extension at 72 °C. Multiple reactions were carried out in parallel to ensure sufficient material for cloning could be generated and the oligos harbored BsaI overhangs for seamless DNA assembly.

PCR products were purified using NucleoSpin Gel and PCR Cleanup columns (Macherey–Nagel). Purified vector DNA (5 µg) and library (1 µg) DNA were digested with *Bsa*I (NEB) and *Dpn*I (NEB) for 3 h at 37 °C in multiple parallel 100 µL reactions and again purified. Vector and library were ligated (1:3 molar ratio, 1 µg total DNA) with NEB T4 DNA ligase for 2 min at 37 °C, followed by 6 h at 25 °C and overnight at 16 °C. DNA was isolated by phenol–chloroform and ethanol-precipitated. Ligated DNA was resuspended in 5 µL ddH_2_O and transformed by electroporation into NEB 10-beta cells.

### Selection

Transformed libraries were plated on LB medium supplemented with suitable ceftazidime concentrations for selection, and incubated at 37 °C overnight. Colonies were harvested with a cell scraper, transferred to 10 ml LB medium containing ceftazidime, and incubated at 37 °C for 2–3 h. The liquid culture was split in three aliquots. One was supplemented with glycerol [to a final 20% (v/v) concentration], and flash frozen for − 80 °C storage. A second was plated on LB medium containing higher ceftazidime concentrations to isolate the most active TEM-1 variants. The remainder was used for plasmid extraction.

Having isolated PTX7 from a stringent selection (300 µg/mL ceftazidime) our second round was carried out at higher stringency (500 µg/mL). PTX8 was the only isolate from that selection (shown in Fig. 2b). Further characterisation of the variant, identified that the plasmid harbouring PTX8 had acquired a mutation that increased its copy number and presumably increased levels of beta-lactamase in the host. Recloning the gene for PTX8 in a naïve plasmid backbone confirmed that finding and it is the recloned gene (therefore assumed to have similar plasmid copy number to other constructs) that has been characterised in Figs. 2 and SI Fig. 4.

### Antibiotic susceptibility assays

The substrate spectrum of TEM-1 variants was tested by measuring the minimum antibiotic concentration that could inhibit bacterial growth in liquid culture (MIC) and by measuring the growth inhibition of bacteria on solid media. *E. coli* harboring TEM-1 variants were tested for their susceptibility against ampicillin (AMP), carbenicillin (CBN), ceftazidime (CAZ), cefotaxime (CTX) and imipenem (IMP).

For MIC determination, approximately 100 CFU (based on the dilution of a liquid culture in mid-log growth) were added to 200 µL LB medium supplemented with different antibiotic concentrations and allowed to grow overnight at 37 °C with shaking. MIC assays were carried out in 96-well flat bottom plates (Greiner). Cells were resuspended by mixing with a multichannel pipette and bacterial growth estimated from OD_600_ measurements. No antibiotic controls were used to estimate the maximum growth of each strain in the experimental conditions and normalize OD_600_ between independent experiments. Growth inhibition assays in liquid cultures were carried out in triplicate with the lowest concentration of the antibiotic to fully inhibit bacterial growth defining MIC for that strain.

Growth inhibition of the *E. coli* strains in solid medium was carried out by placing filter paper discs (Oxoid) containing a known amount of each antibiotic onto a lawn of approximately 10^7^ CFU. Antibiotic susceptibility was measured as the radius of growth inhibition around the antibiotic disc. At least three independent experiments were carried out for each strain.

### DNA library preparation for next generation sequencing (NGS)

Libraries for Illumina MiSeq sequencing were prepared by PCR with oligos containing required adaptors and unique indices to allow all pre- and post-selection libraries to be sequenced in a single experiment.

Pre-selection libraries were amplified directly from the streptavidin beads isolated from assembly. Post-selection libraries were amplified from purified plasmid DNA extracted from recovered transformants. Libraries were amplified in 50 µL reactions using NEB Q5 Hot-start DNA polymerase to minimize amplification errors and PCR cycles capped at 20 to minimize amplification biases. Reactions contained 1 U polymerase, 0.2 µM each of oligos xxx-MiSeqF (separate oligo for each library, with varying index sequences for demultiplexing, names and sequences are in Supplementary Table 4) and TEM1-MiSeq-R, 1 ng plasmid template or 1 µL resuspended bead slurry from the assembled library, 200 µM dNTPs, 1× Q5 reaction buffer, and 1× CES enhancer solution^31^. Product size and purity were checked on agarose gels and correct amplicons excised and purified using Monarch Gel Extraction (NEB).

Libraries were quantified by fluorimetry using a Qubit 3.0 (Thermo Fisher Scientific) with a dsDNA HS assay kit and pooled proportionally to obtain the desired number of reads for each sample. Sequencing was carried out on an Illumina MiSeq instrument by UCL Genomics using a 150 cycle v3 kit.

### NGS data handling

Sequencing data was treated as described in Supplementary Note 2. Briefly, sequences were filtered for quality, trimmed to keep only the diversified regions, translated into protein sequences, counted, and formatted to serve as input for the *k*-mer analysis.

### NGS analysis

Sequencing was modelled as Poisson distributions, to allow different populations to be compared and enrichment of individual sequences determined. All analyses were carried out in MATLAB (MathWorks). A Z-score, defined in (1) was used as a measure of comparison between pre- and post-selection distributions.

1 $$Z = \frac{{\left( {cX - Y} \right) - \left( {c\theta_{X} - \theta_{Y} } \right)}}{{\sqrt {c^{2} \theta_{X} + \theta_{Y} } }}$$

where c is the ratio in size between post- and pre-selection libraries (to correct for sampling), X is the number of counts for a test sequence in the post-selection library, Y is the number of counts for the same sequence in the pre-selection library. θ_X_ and θ_Y_ are the estimated Poisson parameters (counts as fraction of the total reads) for post- and pre-selection libraries respectively. Z-scores give a measure of enrichment, with extremely positive values identifying the sequences most enriched.

Each sequence was decomposed into all possible masked 3-mers and the library termini encoded as “*Z*” characters (to avoid confusion with natural amino acids and degenerate positions). Masked 3-mers were counted and mapped to a 882-dimension column vector, which each dimension representing one of the possible masked 3-mers. Vectors were normalized and scaled by their Z-score.

Once all sequences identified in selection were assembled in column vectors, primary component analysis (PCA) was carried out to identify dimensions (i.e. masked 3-mers) that contributed the most to selection. Sequence reconstruction was carried out for each of the PCA dimensions using positive components above 0.1 (arbitrarily chosen to minimize noise). Reconstruction was carried out by manual inspection assembling selected sequences from the highest to the lowest PCA coefficient. Reconstruction was successful in most cases generating motifs that encompassed both N- and C-terminal arbitrary “Z” characters.

### Loop residue labelling

Numbering of residues within the loop follow the convention for numbering antibody variable regions^40^. Briefly, numbering maintains residues outside the diversified region with their wild-type numbering. Thus, numbering is unchanged for variants of the same length as the wild-type sequence of TEM-1. For variants shorter than wild-type, our scheme introduces a gap between the last diversified codon and downstream invariant position (Table 3).

**Table 3:** Examples of the proposed numbering scheme for length-variant regions.

Length	Variable region	Downstream invariant residue
= WT	_164_RYYGE_168_	_169_L
< WT	_164_RYGT_167_	_169_L
> WT	_164_RGYMKER_168b_	_169_L

Crucially, longer variants are treated as insertions at the end of the diversified region and labelled with an additional lower case letter (e.g. 168a rather than 169) to maintain the residue numbering of the downstream sequence. The proposed numbering scheme unambiguously identifies positions in the sequence without disrupting comparisons between conserved sequence elements outside the library.

## Supplementary Information

Supplementary Information

## Data Availability

The NGS data generated in this study are available from the corresponding author on reasonable request.

## Code and script availability

The MATLAB code for the binomial model used to simulate library assembly is provided as Supplementary Information. Additional MATLAB code and PERL scripts written for sequence analysis are available at https://github.com/PinheiroLab/InDel and from the corresponding author on reasonable request.
