# Supplementary material for: A novel framework for engineering protein loops exploring length and compositional variation
Source: Sci Rep. 2021 Apr 28;11:9134. doi: 10.1038/s41598-021-88708-4 (PMC8080606; doi:10.1038/s41598-021-88708-4)
Supplement: Supplementary file 1 — Supplementary Information [file 41598_2021_88708_MOESM1_ESM.pdf]

Supplementary information

Title (100 characters)

A novel framework for engineering protein loops exploring length and compositional variation.

Authors:

Pedro A. G. Tizei[1], Emma Harris[2], Shamal Withanage[3], Marleen Renders[3] and Vitor B. Pinheiro[1,2, 3*]

Affiliation:

[1] University College London, Department of Structural and Molecular Biology, Gower Street, London, WC1E 6BT, UK.

[2] Birkbeck, Department of Biological Sciences, University of London, Malet Street, WC1E 7HX, UK.

[3] KU Leuven, Rega Institute for Medical Research, Medicinal Chemistry, Herestraat 49 – Box 1041, 3000 Leuven, Belgium.

*corresponding author – v.pinheiro@kuleuven.be

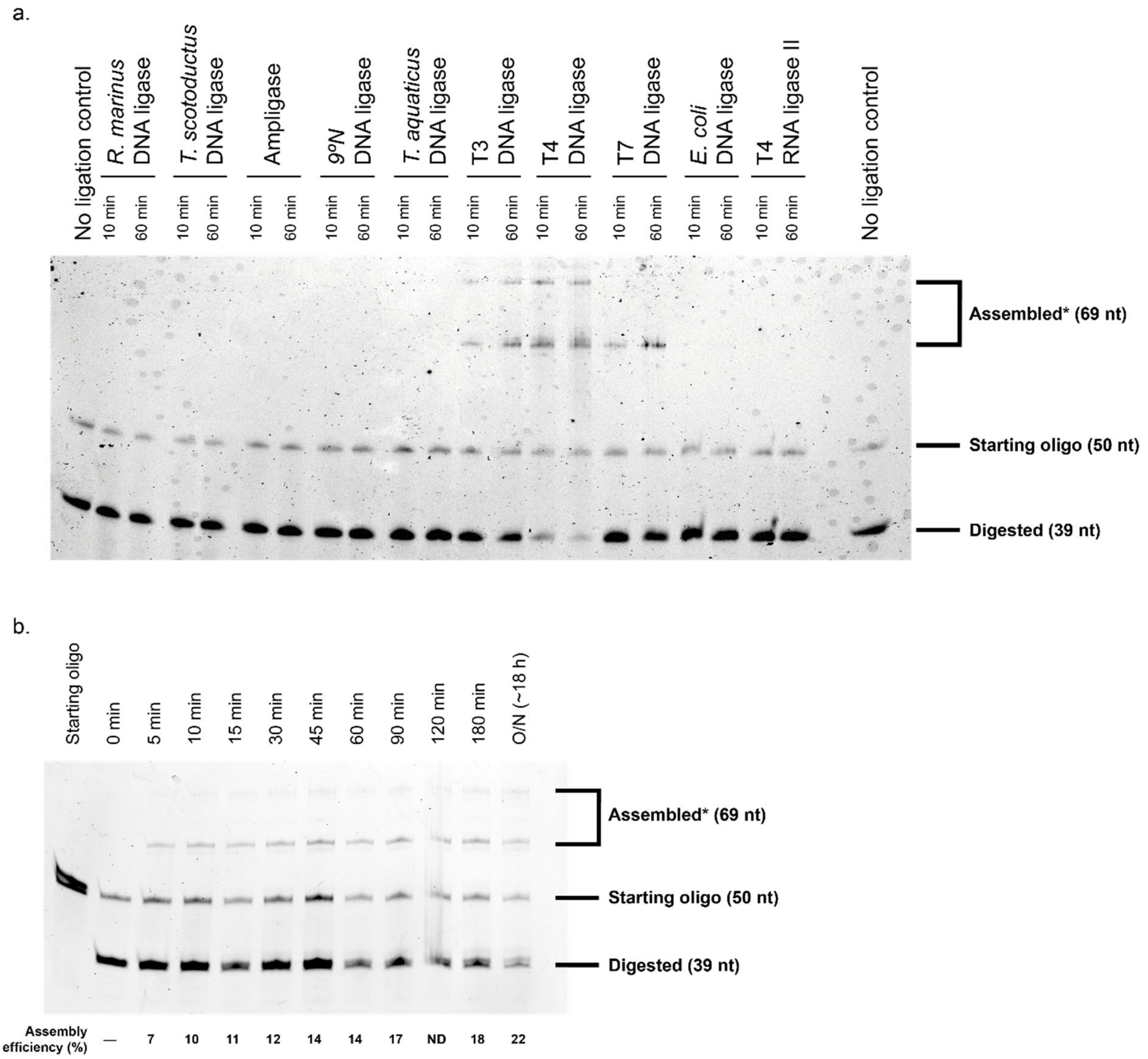

Supplementary Figure 1 Optimization of on-bead ligation. (**a**) Commercially available ligases were tested for efficient DNA ligation of dsDNA template and assembly block. Reaction time is shown and remaining reaction conditions are described in Supplementary Methods. Based on the signal of the ligated product and the decrease in digested fragment, T4 DNA ligase was selected for further optimization. (**b**) Ligation time-course of dsDNA template and assembly block. The dsDNA templates used in these experiments were not phosphorylated. The ligated assembly block (*) commonly migrated as a doublet in experiments using paramagnetic beads. Assembly efficiency, calculated by densitometry as the fraction of assembled product, considered both assembled products and is shown below respective lanes. The sample at 120 min showed significant smearing and was not included in the analysis (ND).

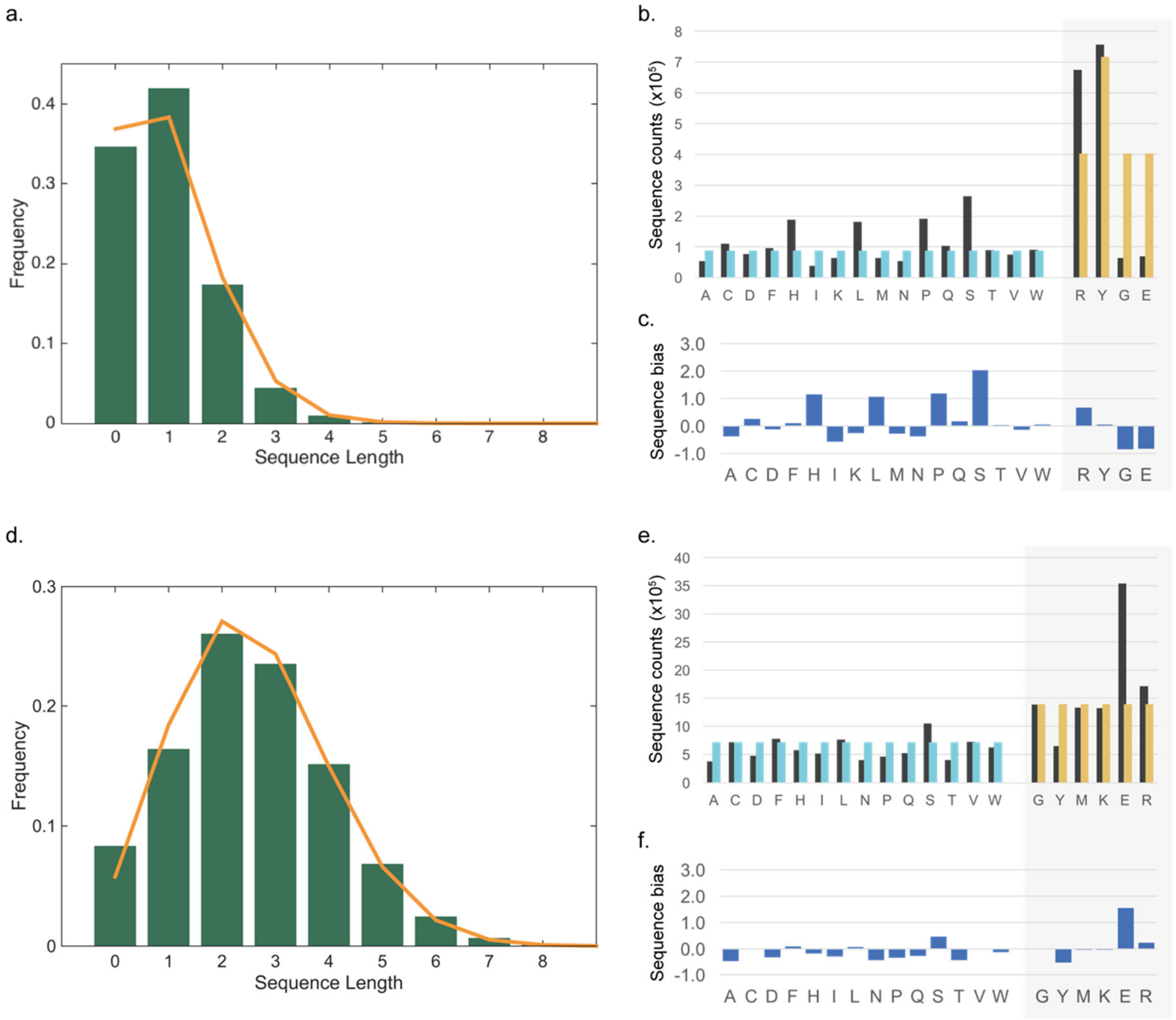

Supplementary Figure 2 InDel assembly efficiency and biases in libraries pre-selection. (**a**,**b**,**c**) refer to the first round library and (**d**,**e**,**f**) refer to the second round library. (**a**,**d**) Library length distribution determined by next generation sequencing (green) and best-fit binomial distribution (orange) used to estimate assembly efficiency per cycle – see supplementary note 1 for details of how that was estimated. Sequence length represents the number of building blocks incorporated in a given construct. Since all building blocks used for the 2 assemblies introduced 3 nucleotides (one codon), the length also represents the number of amino acids encoded in the library. (**b**,**e**) Predicted and observed residue counts. Residues that could only have been incorporated through assembly cycles that included sequence degeneracy (i.e. X in the assemblies in Figure 2b), are shown in cyan. Residues that could be incorporated from degenerate as well as targeted cycles (i.e. the specified residues included as 50% of the assembly mixture – see Figure 2b) are shown in orange. Observed counts are shown in black. (**c**,**f**) Biases of incorporation per amino acid are calculated as $\frac{Observed - Predicted}{Predicted}$. Although biases are present in each library, analysis suggests that there are no systematic strong biases for incorporation.

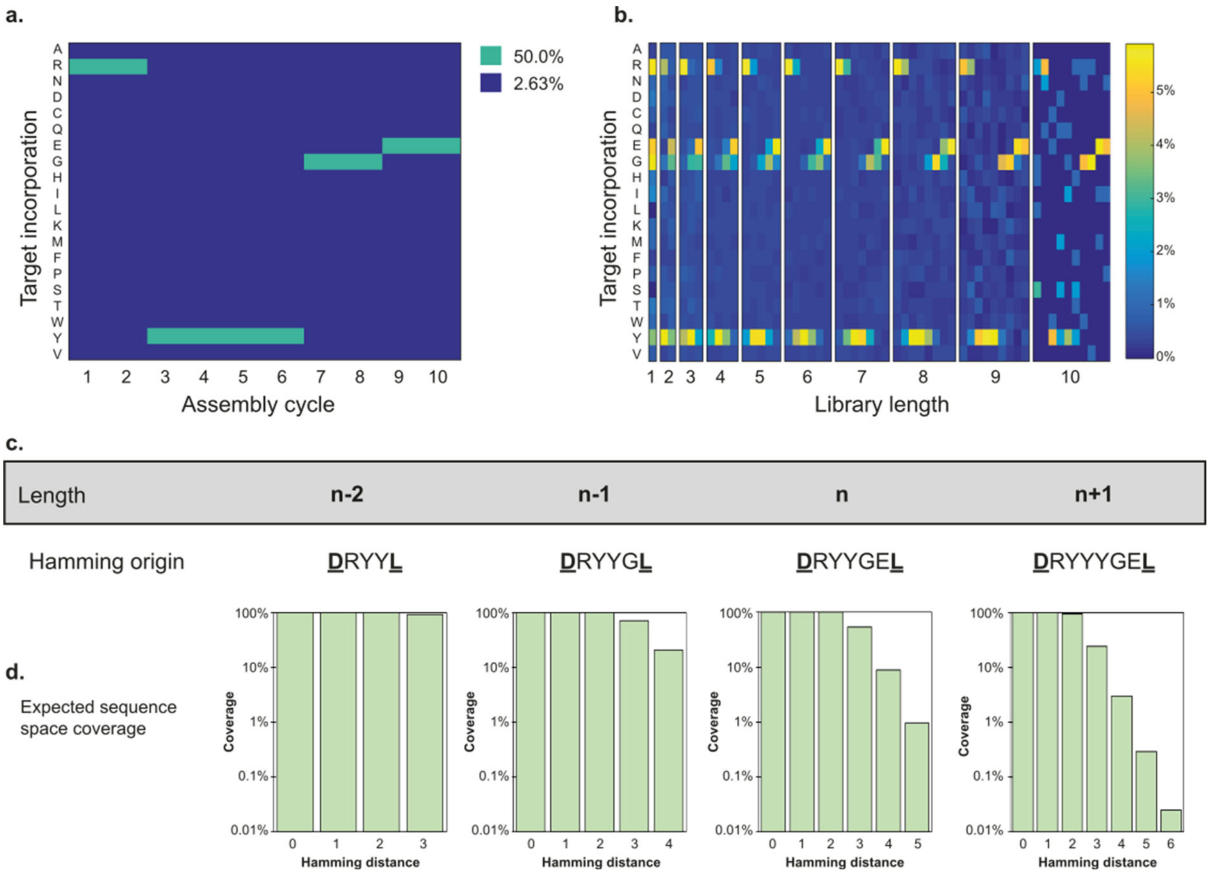

Supplementary Figure 3 Simulation of InDel library assembly. (**a**) First round assembly strategy used in simulation – as per Fig. 2b. (**b**) Based on the 50% incorporation efficiency per cycle, a 10^6 library was generated. Coverage becomes increasingly sparse as the assembled length rises but it is always biased towards R, Y, G and E that were overrepresented in assembly. (**c**) Despite the bias, a 10^6 library under those conditions fully samples sequence spaces of up to 3 insertions, and sample longer landscapes at increasingly sparse coverage – always biased towards the sequence neighbourhood of the target RYYGE motif.

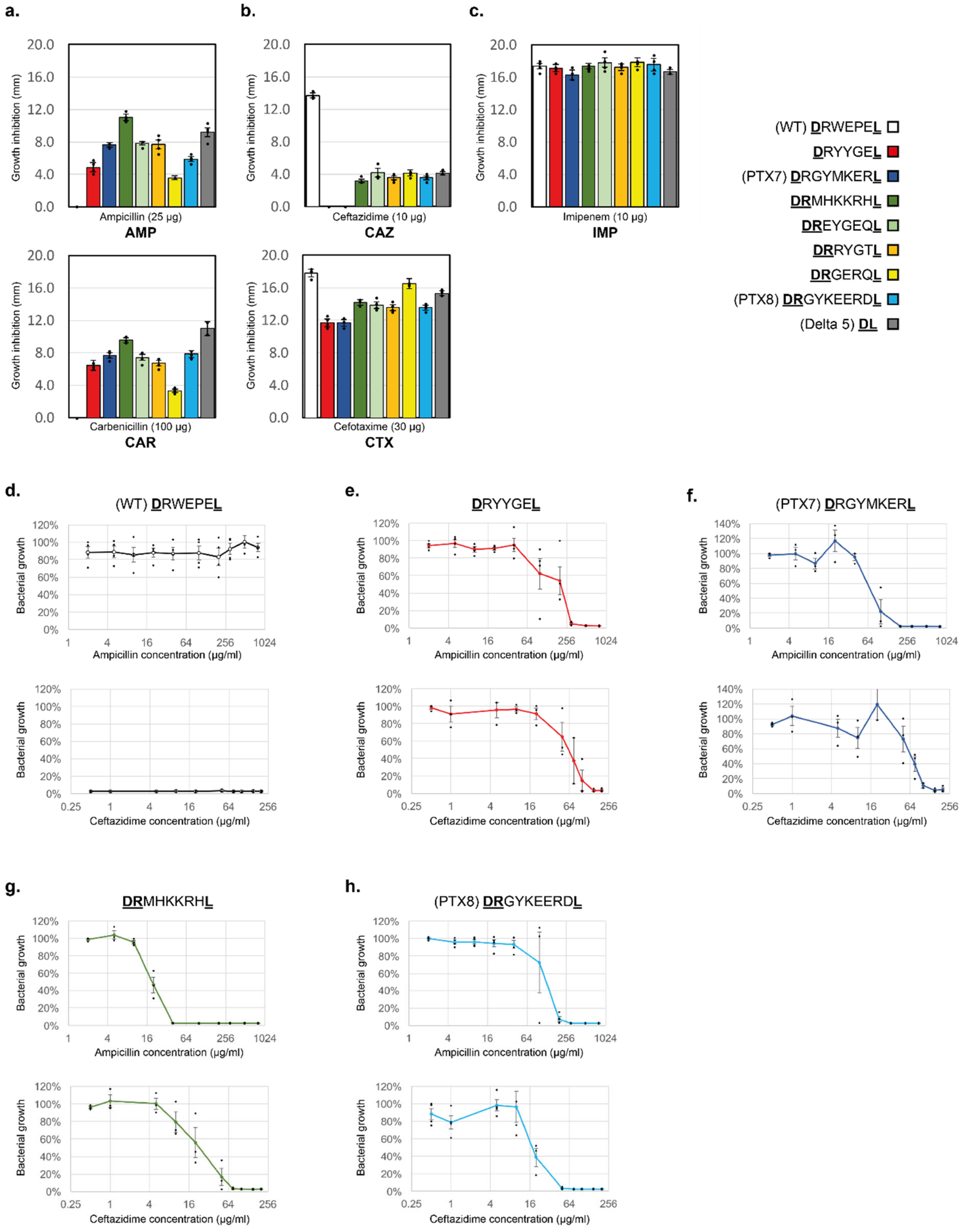

Supplementary Figure 4 Substrate spectrum of TEM-1 variants. Wild-type and engineered TEM-1 were characterized for their antibiotic resistance profile based on the radius of growth inhibition in solid (**a-c**) or liquid (**d-h**) cultures. All strains were characterized for their resistance against (**a**) penams ampicillin (AMP) and carbenicillin (CBN), against (**b**) cephalosporins ceftazidime (CAZ) and cefotaxime (CTX), and against (**c**) carbapenem imipenem. As

previously reported, all engineered lactamases had reduced resistance against penams but were significantly more resistant against cephalosporins. Liquid cultures were used to determine AMP and CAZ minimal inhibitory concentrations (MIC) for selected TEM-1 variants: (**d**) wild-type TEM-1, (**e**) YYGE, (**f**) PTX7, (**g**) MHKKRH - the most enriched sequence after the second round of selection, and (**h**) PTX8. A_{600} of cultures carried out in 96-well plates were normalized against no antibiotic controls. Experiments were carried out in triplicate, individual results are shown (•), as well as average (lines) and standard error of the means. RYYGE MIC_{CAZ} was slightly higher (150 µg/mL) than PTX7 MIC_{CAZ} (100 µg/mL).

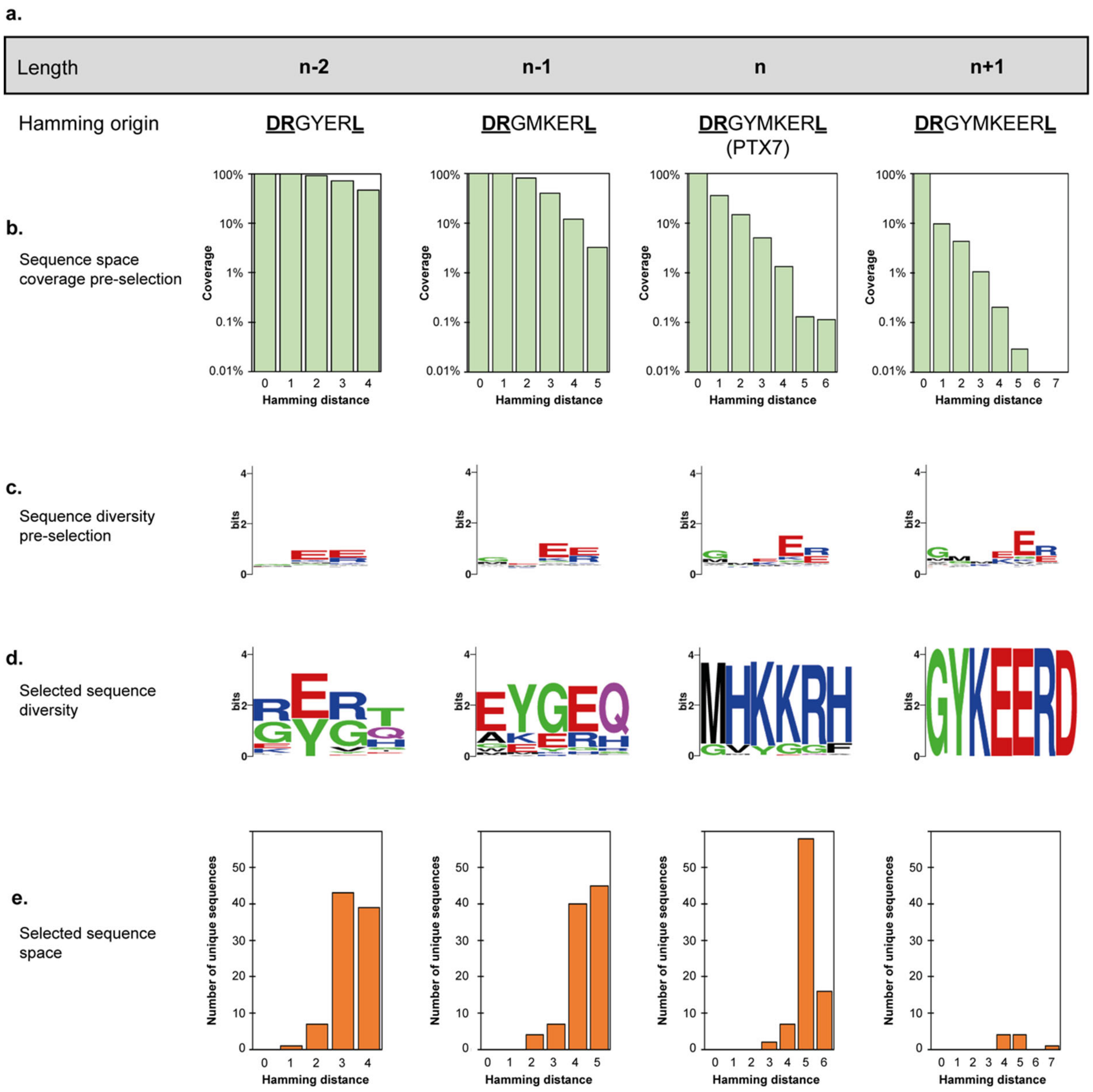

Supplementary Figure 5 InDel assembly coverage of sequence space neighboring PTX7 and impact of selection. (**a**) The available sequence space is split into fixed-length landscapes and each analyzed separately using the most frequent PTX7-related variant of the desired length as the origin for Hamming distances. (**b**) The biased synthesis used in the InDel assembly of this second library ensured the sequence neighborhood of the target GYMKER sequence was efficiently explored, (**c**) with only minimal bias for glutamate in one of the final assembly cycles. The height of each residue in the logo is a measure of their frequency at that position. (**d**) Selection clearly enriches for functional sequences that include RXYG of RYYGE in the ***n-2*** and ***n-1*** landscapes but sample a very diverse and unrelated in the PTX7 landscape. (**e**) Hamming distances to other unique sequences obtained in each landscape after selection.

PCA dimension	PCA-derived motif	Most frequent match	Ranking
1	**Z**(D/T/P)**Z**	**Z**D**Z**<br>**Z**T**Z**<br>**Z**P**Z**	1<br>2<br>3
2	**Z**R(Y/G)YG**Z**<br>**Z**R(Y/G)YGX**Z**<br>**Z**R(Y/G)Y**Z**	**Z**RYYG**Z**<br>**Z**RYYGE**Z**<br>**Z**RYY**Z**	14<br>16<br>324
3	**Z**D**Z**	**Z**D**Z**	1
4	**Z**(D/S)**Z**<br>**Z**YS**Z**	**Z**D**Z**<br>**Z**S**Z**<br>**Z**YS**Z**	1<br>4<br>5
5	**Z**P**Z**	**Z**P**Z**	3
6	**Z**S**Z**<br>**Z**G(G/S)**Z**<br>**Z**GGX**Z**	**Z**S**Z**<br>**Z**GG**Z**<br>**Z**GGW**Z**	4<br>12<br>45
7	**Z**(S/Q)**Z**<br>**Z**(R/S)YYG<br>**Z**YYG	**Z**S**Z**<br>**Z**Q**Z**<br>**Z**RYYGE**Z**<br>**Z**SYYG**Z**<br>**Z**YYGH**Z**	4<br>7<br>16<br>225<br>4568
8	**Z**S**Z**<br>**Z**RGY…**Z**<br>**Z**XH**Z**	**Z**S**Z**<br>**Z**RGYH**Z**<br>**Z**PH**Z**	4<br>10<br>18
9	**Z**(S/G/N)(Y/H/G)**Z**<br>**Z**XYY(G/H)**Z**<br>**Z**(S/G/N)YH**Z**	**Z**GG**Z**<br>**Z**RYYG**Z**<br>**Z**SYH**Z**	12<br>14<br>31
10	**Z**(Q/M)**Z**<br>**Z**RGYX**Z**	**Z**Q**Z**<br>**Z**RGYH**Z**	6<br>10

Supplementary Table 1: Comparison of PCA-derived enriched sequences and NGS read frequency for the 1st round library. Motifs were reconstructed for the first 10 PCA dimensions and used to search the NGS results for the ranking of the highest enriched sequences (highest Z-scores). Although there is a correlation between enrichment, PCA and read frequency (not shown), PCA can identify motifs shorter than a full assembly (e.g. **Z**YYG in PCA_7) and non-conserved residues (e.g. **Z**R(Y/G)YGX**Z** in PCA_2).

PCA component	Masked 3-mer	PCA values	Reconstruction
**1**	**Z**D_'	0.263	
	D**Z**_'	0.275	
	**Z**_**Z**'	0.835	
**Contribution**	**Z**T_'	0.180	
11.19	T**Z**_'	0.182	
**Cumulative**	**Z**P_'	0.175	
11.19	P**Z**_'	0.181	
			**Z**(D/T/P)**Z**
**2**	**Z**R_'	0.503	
	**Z**_Y'	0.176	
	'RY_'	0.228	
	'R_Y'	0.440	
	'YY_'	0.167	
	'Y_G'	0.163	
**Contribution**	**Z**_G'	0.116	
7.63	'YG_'	0.385	
**Cumulative**	G_**Z**'	0.362	**Z**R(G/Y)Y**Z**
18.82	Y_**Z**'	0.163	**Z**R(G/Y)YGX**Z**
			**Z**R(Y/G)YG**Z**
**3**	D**Z**_'	0.579	
	**Z**D_'	0.499	
	'YS_'	-0.100	
	'ZY_'	-0.159	
	'Y_Z'	-0.177	
	'SZ_'	-0.189	**Not detected:**
**Contribution**	'ZT_'	-0.203	Z_Z
4.34	'TZ_'	-0.210	
**Cumulative**	'ZP_'	-0.272	
23.16	'PZ_'	-0.314	
			**Z**D**Z**
**4**	**Z**Y_'	0.452	
	**Z**D_'	0.149	
	D**Z**_'	0.185	
	**Z**S_'	0.132	
	**Z**_S'	0.260	
	'YS_'	0.256	
	Y_**Z**'	0.528	
	S**Z**_'	0.373	**Not detected:**
	'PZ_'	-0.109	Z_Z
**Contribution**	'ZP_'	-0.121	
4.17	'G_Z'	-0.134	
**Cumulative**	'ZT_'	-0.148	
27.33	'TZ_'	-0.156	**Z**(D/S/Y)S**Z**
			**Z**(D/S)**Z**

**5**	'ZP_'	0.448	
**Contribution**	PZ_'	0.525	**Not detected:**
3.69	'ZT_'	-0.482	Z_Z
**Cumulative**	'TZ_'	-0.502	
31.02			**Z**P**Z**
**6**	**Z**G_'	0.283	
	**Z**S_'	0.125	
	S**Z**_'	0.180	
	**Z**_G'	0.383	
	'RG_'	0.128	
	'GG_'	0.174	**Not detected:**
	G_**Z**'	0.496	Z_Z
	G**Z**_'	0.145	
	'PZ_'	-0.106	
	'ZR_'	-0.108	
	'R_Y'	-0.120	
	'Y_G'	-0.128	
**Contribution**	'Y_Z'	-0.189	
2.74	'YY_'	-0.228	
**Cumulative**	'RY_'	-0.264	
33.76	'Z_Y'	-0.322	**Z**GG(X)**Z**
			**Z**(G/S)**Z**
**7**	**Z**S_'	0.398	
	**Z**_**Z**'	0.121	
	S**Z**_'	0.466	
	**Z**_Y'	0.114	
	'RY_'	0.143	
	'YY_'	0.113	
	'Y_G'	0.106	
	Q**Z**_'	0.116	
	'Z_H'	-0.106	**Not detected:**
	'GZ_'	-0.114	GQ_, G_Z, ZR_
	'GY_'	-0.120	
	'ZP_'	-0.132	
	'DZ_'	-0.134	
	'PZ_'	-0.148	
	'ZY_'	-0.163	
	'RG_'	-0.163	
	'ZT_'	-0.201	
**Contribution**	'HZ_'	-0.222	
2.65	'TZ_'	-0.224	
**Cumulative**	'Y_Z'	-0.236	
36.41	'Z_G'	-0.286	**Z**(S/R/X)YYG(S/Q)**Z**
			**Z**(S/Q)**Z**
**8**	**Z**S_'	0.187	
	**Z**_H'	0.246	
	**Z**R_'	0.349	

	'RG_'	0.158	
	'R_Y'	0.171	
	'GY_'	0.102	
	H**Z**_'	0.416	
	S**Z**_'	0.246	
	'PZ_'	-0.109	
	'RY_'	-0.128	**Not detected:**
	'YG_'	-0.165	Z_Z, Z_G
	'GZ_'	-0.167	
	'ZG_'	-0.173	
	'ZY_'	-0.178	
**Contribution**	'Y_G'	-0.196	
2.41	'YY_'	-0.197	
**Cumulative**	'G_Z'	-0.231	**Z**XH**Z**
38.82	'Z_Y'	-0.231	**Z**S**Z**
			**Z**RGY…**Z**
**9**	**Z**G_'	0.154	
	**Z**N_'	0.126	
	'YH_'	0.125	
	'Y_G'	0.187	
	G**Z**_'	0.120	
	N_**Z**'	0.105	
	'Z_M'	-0.102	
	'RR_'	-0.105	**Not detected:**
	'TZ_'	-0.109	Z_Z, HG_
	'Z_R'	-0.111	
	'PZ_'	-0.124	
	'Z_S'	-0.134	
**Contribution**	'YS_'	-0.140	
2.21	'ZY_'	-0.164	
**Cumulative**	'ZR_'	-0.189	**Z**NX**Z**
41.03	'R_Y'	-0.194	**Z**G**Z**
			XYHG**Z**
**10**	'ZM_'	0.114	
	'MZ_'	0.119	
	'GY_'	0.116	
	'PZ_'	-0.110	
	'DZ_'	-0.132	
	'HZ_'	-0.133	
	'ZT_'	-0.138	
	'Z_H'	-0.140	
**Contribution**	'TZ_'	-0.154	
1.91	'G_Z'	-0.164	
**Cumulative**	'SZ_'	-0.198	
42.94	'ZP_'	-0.205	**Z**MGYM**Z**
			**Z**M**Z**

Supplementary Table 2: Reconstructing enriched motifs in the 1st round of selection. Pre- and post-selection library sequencing is used to score sequences based on their enrichment in the population. Sequences are then decomposed using the masked k-mer analysis and principal component analysis (PCA) carried out to reconstruct enriching motifs in the library - as described in Figure 4. Masked 3-mers identified as enriching (i.e. positive PCA score greater than 0.05) are used to reconstruct the motifs being selected, with depleted 3-mers (i.e. negative PCA scores) used to avoid false positives. We show here the first 10 PCA components (assembled in SI Table 1) and most significant 3-mer scores (|score|>0.1). Enriching 3-mers are presented broadly in reconstruction order to facilitate interpretation, while negative PCA values are shown in order. Where ambiguity in assembly was observed (e.g. 1st component), all possible variants were considered. Missing k-mers (i.e. not detected above cut-offs) are also highlighted.

PCA component	Masked 3-mer	PCA values	Reconstruction
**1**	'**Z**M_'	0.271	
	'**Z**_H'	0.27	
	'MH_'	0.27	
	'M_K'	0.271	
	'HK_'	0.27	
	'H_K'	0.267	
	'KK_'	0.267	
	'K_R'	0.29	
	'KR_'	0.267	
**Contribution**	'K_H'	0.267	
30.77	'RH_'	0.289	
**Cumulative**	'R_**Z**'	0.305	
30.77	'H**Z**_'	0.291	
			**Z**MHKKRH**Z**
**2**	'**Z**E_'	0.269	
	'**Z**_Y'	0.36	
	'EY_'	0.267	
	'E_G'	0.265	
	'YG_'	0.348	
	'Y_E'	0.27	
	'GE_'	0.306	
**Contribution**	'G_Q'	0.268	
19.37	'EQ_'	0.263	
**Cumulative**	'E_**Z**'	0.269	
50.14	'Q**Z**_'	0.308	
			**Z**EYGEQ**Z**
**3**	'**Z**R_'	0.308	
	'**Z**_Y'	0.224	
	'RY_'	0.308	
	'R_G'	0.308	
	'YG_'	0.234	
	'Y_T'	0.308	
	'GT_'	0.307	
	'G_**Z**'	0.33	
	'T**Z**_'	0.308	
	'R_Z'	-0.103	
	'G_Q'	-0.103	
	'ZE_'	-0.105	
**Contribution**	'ER_'	-0.116	
10.49	'Z_E'	-0.129	
**Cumulative**	'QZ_'	-0.186	
60.62	'GE_'	-0.187	
			**Z**RYGT**Z**
**4**	'**Z**G_'	0.396	

	'**Z**_E'	0.351	
	'GE_'	0.131	
	'G_R'	0.263	
	'G_**Z**'	0.12	
	'ER_'	0.35	
	'E_Q'	0.229	
	'RQ_'	0.255	
	'R_**Z**'	0.316	
	'Q**Z**_'	0.126	
	'Y_E'	-0.114	
	'G_Q'	-0.115	
	'EY_'	-0.123	
**Contribution**	'ZE_'	-0.125	
8.26	'E_G'	-0.133	
**Cumulative**	'EQ_'	-0.134	
68.88	'E_Z'	-0.139	**Z**GERQ**Z**
			**Z**GE**Z**
**5**	'**Z**G_'	0.378	
	'**Z**_V'	0.332	
	'GV_'	0.332	
	'G_Y'	0.358	
	'VY_'	0.331	
	'V_G'	0.157	
	'V_**Z**'	0.169	
	'Y**Z**_'	0.175	
	'Y_G'	0.135	
	'GG_'	0.135	**Not detected:**
	'G_F'	0.135	G_Z, YG_
	'GF_'	0.135	
**Contribution**	'F**Z**_'	0.134	
5.24	'Z_E'	-0.132	
**Cumulative**	'R_Z'	-0.143	
74.12	'ER_'	-0.174	**Z**GVY**Z**
			**Z**GVYGGF**Z**
**6**	'**Z**A_'	0.212	
	'**Z**_K'	0.201	
	'AK_'	0.194	
	'A_E'	0.21	
	'KE_'	0.291	
	'K_R'	0.19	
	'ER_'	0.147	
	'E_H'	0.358	
	'RH_'	0.187	
	'H**Z**_'	0.277	
	'**Z**E_'	0.133	
	'**Z**_V'	0.118	
	'GV_'	0.118	

	'G_Y'	0.116	
	'VY_'	0.118	
	'V_**Z**'	0.15	
	'G_V'	-0.103	**Not detected:**
	'VZ_'	-0.104	ZG_, EV_, EK_,
	'YV_'	-0.104	R_Z, YZ_
	'Y_Z'	-0.112	
	'GE_'	-0.113	
	'GY_'	-0.12	
	'QZ_'	-0.134	
**Contribution**	'ZG_'	-0.147	
3.73	'E_Q'	-0.159	
**Cumulative**	'G_R'	-0.161	GVY**Z**
77.85	'RQ_'	-0.174	**Z**AKERH**Z**
			**Z**E(V/K/R)X**Z**
**7**	'**Z**G_'	0.217	
	'**Z**_Y'	0.366	
	'GY_'	0.374	
	'G_V'	0.312	
	'YV_'	0.313	
	'Y_**Z**'	0.321	
	'V**Z**_'	0.312	
	'**Z**A_'	0.107	
	'A_E'	0.107	**Not detected:**
	'KE_'	0.123	AK_, HZ_
	'E_H'	0.107	
	'YG_'	-0.114	
**Contribution**	'Z_E'	-0.116	
3.46	'G_Z'	-0.124	
**Cumulative**	'RQ_'	-0.132	
81.3	'QZ_'	-0.154	**Z**(A/G)YV**Z**
			**Z**AKEXH
**8**	'**Z**E_'	0.277	
	'**Z**_E'	0.359	
	'EE_'	0.293	
	'E_V'	0.273	
	'EV_'	0.281	
	'E_H'	0.159	
	'VH_'	0.274	
	'V_**Z**'	0.354	
	'H**Z**_'	0.191	
	'FZ_'	-0.101	
	'K_R'	-0.123	
	'Z_K'	-0.124	
	'AK_'	-0.129	
	'R_Z'	-0.141	
**Contribution**	'KE_'	-0.147	

2.23	'ER_'	-0.156	
**Cumulative**	'A_E'	-0.164	
83.54	'ZA_'	-0.168	
			**Z**EEVH**Z**
**9**	'**Z**W_'	0.203	
	'**Z**_E'	0.2	
	'WE_'	0.203	
	'W_G'	0.203	
	'V_G'	0.113	
	'EG_'	0.266	
	'E_R'	0.472	
	'GR_'	0.266	
	'R**Z**_'	0.195	
	'G_Q'	0.163	
	'RQ_'	0.116	
	'**Z**A_'	0.112	
	'**Z**_Y'	0.11	
	'AY_'	0.136	
	'YE_'	0.17	
	'Y_H'	0.152	
	'EH_'	0.152	**Not detected:**
	'HR_'	0.136	G_Z, A_E, R_Z, QZ_
	'H_**Z**'	0.136	
	'E_Q'	-0.11	
**Contribution**	'ER_'	-0.119	
1.71	'ZG_'	-0.122	
**Cumulative**	'G_R'	-0.153	
85.25	'GE_'	-0.205	**Z**AYEHR**Z**
			**Z**WERGR(Q)**Z**
			VEGRQ
**10**	'**Z**A_'	0.163	
	'**Z**_Y'	0.112	
	'AY_'	0.139	
	'A_E'	0.162	
	'YE_'	0.234	
	'Y_H'	0.205	
	'EH_'	0.206	
	'HR_'	0.139	
	'H_**Z**'	0.14	
	'R**Z**_'	0.119	
	'**Z**G_'	0.107	
	'G_Y'	0.121	
	'V_**Z**'	0.233	
	'Y**Z**_'	0.258	
	'GY_'	-0.104	
	'YV_'	-0.128	
	'G_V'	-0.128	**Not detected:**

	'VZ_'	-0.128	E_R
	'Y_Z'	-0.138	
	'YG_'	-0.151	
	'G_F'	-0.191	
	'GG_'	-0.192	
	'Y_G'	-0.192	
	'GF_'	-0.192	
**Contribution**	'Z_E'	-0.198	
1.56	'FZ_'	-0.206	VX**Z**
**Cumulative**	'G_Z'	-0.208	**Z**(A/G)Y(Y/E)HR**Z**
86.81	'V_G'	-0.216	**Z**G(Y/X)(Y)**Z**
			**Z**GAYEHR**Z**

Supplementary Table 3: Reconstructing enriched motifs in the 2nd round of selection. Pre- and post-selection library sequencing is used to score sequences based on their enrichment in the population. Sequences are then decomposed using the masked k-mer analysis and principal component analysis (PCA) carried out to reconstruct enriching motifs in the library - as described in Figure 4. Masked 3-mers identified as enriching (i.e. positive PCA score greater than 0.05) are used to reconstruct the motifs being selected, with depleted 3-mers (i.e. negative PCA scores) used to avoid false positives. We show here the first 10 PCA components (assembled in Table 1) and most significant 3-mer scores (|score|>0.1). Enriching 3-mers are presented broadly in reconstruction order to facilitate interpretation, while negative PCA values are shown in order. Where ambiguity in assembly was observed (e.g. 4th component), all possible variants were considered. Missing k-mers (i.e. not detected above cut-offs) are also highlighted.

Name	Sequence (5' → 3')	Use
**InDel Assembly**		
Init-Opt-FAM-T	GTGTGGCGTGTAGGTAAGATGATTTCCTTCA CCTATGATAGAAGAGCAAA	Fluorescent-labelled biotinylated initiation block for assembly condition optimization (5' Biotin-TEG and dT fluorescein at the position in bold)
Init-Opt-B	TTTGCTCTTCTATCATAGGTGAAGGAAATCAT CTTACCTACACGCCACAC	Initiation block for assembly condition opimization
Cap-Opt-T	NNNACCGGTCGTGGTGCTGTTTCTGAAAAAG ATGCGCCAAAAGA	3' Capping block for assembly condition optimization
Cap-Opt-B	TCTTTTGGCGCATCTTTTTCAGAAACAGCACC ACGACCGGT	3' Capping block for assembly condition optimization
Init-TEM1-1G-T	ACCGCTTTTTTGCACAACATGGGGGATCATG TAACTCGCCTTGATAGAAGAGCAAA	Initiation block for 1G library
Init-TEM1-1G-B	TTTGCTCTTCTATCAAGGCGAGTTACATGATC CCCCATGTTGTGCAAAAAAGCGGT	Initiation block for 1G library
Init-TEM1-2G-T	CTTTTTTGCACAACATGGGGGATCATGTAACT CGCCTTGATCGTAGAAGAGCAAA	Initiation block for 2G library
Init-TEM1-2G-B	TTTGCTCTTCTACGATCAAGGCGAGTTACATG ATCCCCCATGTTGTGCAAAAAAG	Initiation block for 2G library
Cap-TEM1-T	NNNCTGAATGAAGCCATACCAAACGACGAGC GTGACACCACGACCCCT	3' Capping block for both diversification rounds
Cap-TEM1-B	AGGGGTCGTGGTGTCACGCTCGTCGTTTGGT ATGGCTTCATTCAG	3' Capping block for both diversification rounds

BB-A-T	NNNGCAAGAAGAGCTCATCCCTGTCTGTATG TC	Building block for Ala
BB-A-B	GACATACAGACAGGGATGAGCTCTTCTTGC	Building block for Ala
BB-C-T	NNNTGCAGAAGAGCTCATCCCTGTCTGTATG TC	Building block for Cys
BB-C-B	GACATACAGACAGGGATGAGCTCTTCTGCA	Building block for Cys
BB-D-T	NNNGACAGAAGAGCTCATCCCTGTCTGTATG TC	Building block for Asp
BB-D-B	GACATACAGACAGGGATGAGCTCTTCTGTC	Building block for Asp
BB-E-T	NNNGAGAGAAGAGCTCATCCCTGTCTGTATG TC	Building block for Glu
BB-E-B	GACATACAGACAGGGATGAGCTCTTCTCTC	Building block for Glu
BB-F-T	NNNTTCAGAAGAGCTCATCCCTGTCTGTATG TC	Building block for Phe
BB-F-B	GACATACAGACAGGGATGAGCTCTTCTGAA	Building block for Phe
BB-G-T	NNNGGTAGAAGAGCTCATCCCTGTCTGTATG TC	Building block for Gly
BB-G-B	GACATACAGACAGGGATGAGCTCTTCTACC	Building block for Gly
BB-H-T	NNNCACAGAAGAGCTCATCCCTGTCTGTATG TC	Building block for His
BB-H-B	GACATACAGACAGGGATGAGCTCTTCTGTG	Building block for His
BB-I-T	NNNATCAGAAGAGCTCATCCCTGTCTGTATG TC	Building block for Ile
BB-I-B	GACATACAGACAGGGATGAGCTCTTCTGAT	Building block for Ile
BB-K-T	NNNAAGAGAAGAGCTCATCCCTGTCTGTATG TC	Building block for Lys
BB-K-B	GACATACAGACAGGGATGAGCTCTTCTCTT	Building block for Lys
BB-L-T	NNNCTGAGAAGAGCTCATCCCTGTCTGTATG TC	Building block for Leu
BB-L-B	GACATACAGACAGGGATGAGCTCTTCTCAG	Building block for Leu
BB-M-T	NNNATGAGAAGAGCTCATCCCTGTCTGTATG TC	Building block for Met
BB-M-B	GACATACAGACAGGGATGAGCTCTTCTCAT	Building block for Met
BB-N-T	NNNAACAGAAGAGCTCATCCCTGTCTGTATG TC	Building block for Asn

BB-N-B	GACATACAGACAGGGATGAGCTCTTCTGTT	Building block for Asn
BB-P-T	NNNCCAAGAAGAGCTCATCCCTGTCTGTATG TC	Building block for Pro
BB-P-B	GACATACAGACAGGGATGAGCTCTTCTTGG	Building block for Pro
BB-Q-T	NNNCAGAGAAGAGCTCATCCCTGTCTGTATG TC	Building block for Gln
BB-Q-B	GACATACAGACAGGGATGAGCTCTTCTCTG	Building block for Gln
BB-R-T	NNNCGTAGAAGAGCTCATCCCTGTCTGTATG TC	Building block for Arg
BB-R-B	GACATACAGACAGGGATGAGCTCTTCTACG	Building block for Arg
BB-S-T	NNNTCCAGAAGAGCTCATCCCTGTCTGTATG TC	Building block for Ser
BB-S-B	GACATACAGACAGGGATGAGCTCTTCTGGA	Building block for Ser
BB-T-T	NNNACCAGAAGAGCTCATCCCTGTCTGTATG TC	Building block for Thr
BB-T-B	GACATACAGACAGGGATGAGCTCTTCTGGT	Building block for Thr
BB-V-T	NNNGTGAGAAGAGCTCATCCCTGTCTGTATG TC	Building block for Val
BB-V-B	GACATACAGACAGGGATGAGCTCTTCTCAC	Building block for Val
BB-W-T	NNNTGGAGAAGAGCTCATCCCTGTCTGTATG TC	Building block for Trp
BB-W-B	GACATACAGACAGGGATGAGCTCTTCTCCA	Building block for Trp
BB-Y-T	NNNTACAGAAGAGCTCATCCCTGTCTGTATG TC	Building block for Tyr
BB-Y-B	GACATACAGACAGGGATGAGCTCTTCTGTA	Building block for Tyr

Cloning

Vec-TEM1-F	ATCGGTCTCAGTAACCCATGCGAGAGTAGGG AACTG	Amplification of the vector for cloning TEM-1 coding sequence
Vec-TEM1-R	ATCGGTCTCATCATGGTTAATTCCTCCTGTTA GCCCAAAAAAC	Amplification of the vector for cloning TEM-1 coding sequence
TEM1-Nter-F	ATCGGTCTCAATGAGTATTCAACATTTCCGTG TCGCC	Amplification of the TEM-1 coding sequence for cloning
TEM1-Nter-R	ATCGGTCTCAGGGACCCACGCTCACCGGC	Amplification of the TEM-1 coding sequence for cloning
TEM1-Cter-F	ATCGGTCTCAUCCCGCGGTATCATTGCAGCA CTG	Amplification of the TEM-1 coding sequence for cloning
TEM1-Cter-R	ATCGGTCTCATTACCAATGCTTAATCAGTGAG GCACCTATC	Amplification of the TEM-1 coding sequence for cloning
TEM1-YYG-F	ATCGGTCTCATATTACGGTGAGCTGAATGAA GCCATACCAAACG	Creating the RYYGE variant as a control
TEM1-MutLoop-R	ATCGGTCTCAAATAACGATCAAGGCGAGTTA CATGATC	Creating the RYYGE variant as a control
TEM1-M182T-F	CCCCTGCAGCAATGGCAACAAC	Inverse PCR primer for introduction of M182T mutation into TEM-1
TEM1-M182T-R	TCGTGGTGTCACGCTCG	Inverse PCR primer for introduction of M182T mutation into TEM-1
TEM1-InDel-AmpF1	ATCGGTCTCAACCGCTTTTTTGCACAACATG G	Amplification of the first round library fragments for cloning into the constant region of TEM-1
TEM1-InDel-AmpF2	ATCGGTCTCACTTTTTTGCACAACATGGGGG ATC	Amplification of the second round library fragments for cloning into the constant region of TEM-1
TEM1-InDel-AmpR	ATCGGTCTCAAGGggTCGTGGTGTCAC	Amplification of library fragments for cloning into the constant region of TEM-1
Vec-TEM1-InDel-F	ATCGGTCTCAcCCTGCAGCAATGGCAACAAC	Amplification of the vector for cloning of library fragments
Vec-TEM1-InDel-R1	ATCGGTCTCACGGTTAGCTCCTTCGGTCC	Amplification of the vector for cloning of first round library fragments
Vec-TEM1-InDel-R2	ATCGGTCTCAAAAGCGGTTAGCTCCTTCGGT CC	Amplification of the vector for cloning of second round library fragments
TEM1-Seq1	GTGAAAGTAAAAGATGCTG	Sequencing primer for construct checking
TEM1-Seq2	AGGCAACTATGGATGAACG	Sequencing primer for construct checking

TEM1-Seq3	GATTTGAACGTTGCGAAG	Sequencing primer for construct checking
TEM1-Seq4	AACGCTCGGTTGCC	Sequencing primer for construct checking
TEM1-Seq5	GAAAACGTTCTTCGGGGC	Sequencing primer for construct checking
TEM1-Seq6	TTTGAGTGAGCTGATACCG	Sequencing primer for construct checking
TEM1-Seq7	GGGATTTTGGTCATGAG	Sequencing primer for construct checking

NGS

TEM1-MiSeq-R	CAAGCAGAAGACGGCATACGAGATCGGTCTC GGCATTCCTGCTGAACCGCTCTTCCGATCTA GGGGTCGTGGTGTCAC	Reverse primer for NGS libraries
1G-Pre-MiSeq-F	AATGATACGGCGACCACCGAGATCTACACTC TTTCCCTACACGACGCTCTTCCGATCTNNNC GATGTACCGCTTTTTTGCACAACATGG	Forward primer for pre-selection first round library
1G-Post-MiSeq-F	AATGATACGGCGACCACCGAGATCTACACTC TTTCCCTACACGACGCTCTTCCGATCTNNNTT AGGCACCGCTTTTTTGCACAACATGG	Forward primer for post-selection first round library
2G-Pre-MiSeq-F	AATGATACGGCGACCACCGAGATCTACACTC TTTCCCTACACGACGCTCTTCCGATCTNNNC AGATCCTTTTTTGCACAACATGGGGGATC	Forward primer for pre-selection second round library
2G-Post-MiSeq-F	AATGATACGGCGACCACCGAGATCTACACTC TTTCCCTACACGACGCTCTTCCGATCTNNNG ATCAGCTTTTTTGCACAACATGGGGGATC	Forward primer for post-selection second round library

Supplementary Table 4 Oligonucleotides used in this work.

Library	Quality filtered count	Matched count
1st round pre-selection	3256942	2269710
1st round post-selection	320891	228691
2nd round pre-selection	7877580	6575072
2nd round post-selection	65064	63612

Supplementary Table 5 Read counts in NGS libraries after quality filtering and after matching translated sequence ends.

Supplementary Note 1 – Distribution of incorporation

Given the stochastic and discrete nature of the InDel cycle, a simple agent-based binomial model was used to simulate the incorporation per cycle of the available building blocks.

For a given agent *i,* $P(x_n \rightarrow x_{n+1}) = p$, where p is the cycle efficiency.

As shown below in the MATLAB code, that simple routine can be applied to each individual agent sequentially. A cycle of InDel assembly represents this binomial simulation applied once to each and every agent available. Successful events lead to the incorporation of the building block, unsuccessful events leave the agent unchanged. Repetition of those cycles can be implemented to simulate the multiple cycles of the assembly process.

Nevertheless, assembly also must consider the identity of the building block being used in that particular cycle. We reasoned that given the large number of agents (and even greater number of molecules in the real assembly) a simple random selection algorithm would be adequate. At this step, if a successful incorporation is detected, the algorithm picks among one of the building blocks used in that particular cycle. The probability of selection is proportional to the molar ratio used in the reaction, i.e. if Ala:Phe blocks are used 1:4, then the probability of incorporating Phe is four times higher than Ala.

This approach assumes no bias in incorporation between the different building blocks. This was expected given the small differences in sequence and length of the blocks used in this report – that is also supported in the analysis of the generated library (Supplementary Fig. 2).

An example of the block distribution per cycle is shown in Supplementary Fig. 4a and the result of the assembly, stratified by length, shown in Supplementary Fig. 4b.

MATLAB script

```
%% InDEL assembly prediction (v.0.2)
% By V. Pinheiro

%% Creating the assembly setup

prompt = ['How many assembly cycles'];
dlg_title = 'Assembly cycles';
num_lines = 1;
cycles = inputdlg(prompt, dlg_title, num_lines);
cycles = str2num(cycles{1});
% Asks user prompt for assembly cycle number

composition = zeros(20,cycles);

for n = 1: cycles;
    prompt = {'A', 'C', 'D', 'E', 'F', 'G', 'H', 'I', 'K', 'L', 'M', 'N',
'P', 'Q', 'R', 'S', 'T', 'V', 'W', 'Y'};
    dlg_title = 'What is your desired composition for this cycle?';
    num_lines = 1;
    def = {'0', '0','0','0','0','0','0','0','0','0','0',
'0','0','0','0','0','0','0','0','0'};
    composition_answer = inputdlg(prompt, dlg_title, num_lines, def);
```

```
    for a = 1: 20;
        composition(aa2int(prompt{a}),n) = str2num(composition_answer{a});
    end
end
% Asks user to choose ratio of amino acids for assembly cycle


composition_freq = zeros(20,cycles);
for n = 1: size(composition, 2);
    total = sum(composition(:,n));
    composition_freq (:,n) = composition (:,n)/total;
end
% Adjusting amino acid frequencies

figure(1);
imagesc(composition_freq, [0,1]);
set(gca,'xtick',[],'ytick',[]); %colormap bone;

%% Generating a population

prob_ligation = 0.5;
n_samples = 10000;
% Defining system variables

lib = zeros(n_samples,cycles);
for n = 1: n_samples;
    for x = 1: cycles;
        a = rand(1);
        if a <= prob_ligation;
            b = rand(1);
            for c = 1: 20;

                if b > 0;
                    lib(n,x) = c;
                    b = b - composition_freq(c,x);
                end
            end
        end
    end
end


% This for loop simulates the assembly method, generating a matrix (lib)
% containing the result of individual simulated assembly experiments.
% Matrix uses the numerical codes for the amino acids in the cycle they are
% incorporated.


%% Collating the resulting libraries

Output = {};

for n = 1: n_samples;
    Clone = [];
    for x = 1: cycles;
        if lib (n,x) ~= 0;
            Clone = horzcat(Clone, int2aa(lib(n,x)));
        end
    end
    Output = vertcat(Output, Clone);
end
```

```
% This for loop converts the sparse simulation matrix into a cell
% containing the resulting 'obtained' library, which is the starting point
% of the analysis and similar to what would be obtained from a deep
% sequencing run.

%% Counting mutants

Freqs = tabulate(Output);
% Make a table with the frequency of each variant

%% Creating a frequency and composition table

Max_column = ((cycles+1)/2)*cycles;
End_column = zeros(cycles, 1);
Start_column = zeros(cycles, 1);
Output_count = zeros (20, Max_column);
Output_frequency = zeros (20, Max_column);

% This creates the basic matrix where all loop lengths will be stored.
% Columns will be sequential so n=1 will be column 1, n=2 will be columns 2
% and 3, and so on. That will be the simplest route to making a master
% table that can be broken into smaller sets.


for n = 1: cycles;
    End_column(n) = ((n+1)/2)*n;
    Start_column(n) = End_column(n) - n +1;
end
% This generates a vector identifying the limits of each sublibrary

for n = 1: length(Output);
    a=0;
    for a = 1: length(Output{n});
        Column = ((length(Output{n})+1)/2)*length(Output{n}) -
length(Output{n}) + a;
        Output_count(aa2int(Output{n}(a)),Column) =
Output_count(aa2int(Output{n}(a)),Column) + 1;
    end
end

% Reads all assembly outputs separating them by length and counting the
% occurences of each amino acid at a given position of the library.

total = zeros(cycles,1);

% Creates a vector containing the sum of sequences for each sublibrary

for n = 1 : cycles;
    total(n) = sum(sum(Output_count(:,Start_column(n):End_column(n))));
    Output_frequency(:,Start_column(n):End_column(n)) =
Output_count(:,Start_column(n):End_column(n))/total(n);
end

% Creates a frequency matrix for each sublibrary

total_frequencies = zeros(cycles,1);
for a = 1: length(total);
    if total(a) ~= 0;
        total_frequencies(a) = total(a)/sum(total);
    else
```

```
        total_frequencies(a) = 0;
    end
end

% Converts the total count into a frequency table

%% Histogram

figure(2);
bar(total_frequencies);

%% Amino acid positional probability per sublibrary

figure(3);

Gap = 0.5;
Left = zeros(cycles, 1);
Width = zeros(cycles, 1);

for n = 1: cycles;
    Left (n) = (Start_column(n) + (n*Gap))/(Max_column + (cycles+2)*Gap);
    Width (n) = (End_column(n) - Start_column (n) +1)/(Max_column +
(cycles+2)*Gap);
end

% This for loop defines the placement for each sublibrary graph

for b = 1: cycles;
    if total(b) ~= 0;
        Max_freq =
max(max(Output_frequency(:,Start_column(b):End_column(b))));
    else
        Max_freq = 1;
    end

    % This if routine normalises the display scale in each sublibrary
    % ensuring that the highest frequency incorporation is used to
    % normalise the displayed signal in each sublibrary

    axes('Position', [Left(b), 0.05, Width(b), 0.75]);
    imagesc(Output_frequency(:,Start_column(b):End_column(b)),
[0,Max_freq]);
    set(gca,'xtick',[],'ytick',[]); %colormap bone;

    axes('Position', [Left(b), 0.85, Width(b), 0.05]);
    imagesc(total_frequencies(b),[0,max(total_frequencies)]);
    set(gca,'xtick',[],'ytick',[]);
end

% This generates a compound figure showing the frequency of the
% sublibraries and the positional composition of each sublibrary.

%% Amino acid positional probability taking the whole population into
consideration

figure(4);

% This requires a new frequency matrix with positional distribution
% calculated for the entire population rather than per sublibrary

Output_frequency_all = Output_count/sum(total);
```

```
Max_freq2 = max(max(Output_frequency_all));

for b = 1: cycles;


    axes('Position', [Left(b), 0.05, Width(b), 0.9]);
    imagesc(Output_frequency_all(:,Start_column(b):End_column(b)),
[0,Max_freq2]);
    set(gca,'xtick',[],'ytick',[]); %colormap bone;

end

% This generates a compound figure showing the frequency of the
% sublibraries and the positional composition of each sublibrary based on
% the total frequency

%% Cleaning up unnecessary variables

varlist = {'Output', 'lib', 'a', 'b', 'c', 'Clone', 'Column',
'composition_answer', 'cycles', 'def', 'dlg_title', 'End_column', 'Gap',
'Left', 'Max_column', 'Max_freq', 'Max_freq2', 'n', 'num_lines', 'prompt',
'total', 'total_frequencies', 'Width', 'x', 'varlist'};
clear(varlist{:});
```

Supplementary Note 2 - NGS Analysis workflow

1- **Trimming** – Remove the initial NNN nucleotides from all sequences and trims them to 100 nucleotide length, which is more than is needed to cover all possible variants for the Ω-loop (fastx_trimmer is from the FASTX-Toolkit, available at: http://hannonlab.cshl.edu/fastx_toolkit/)

```
fastx_trimmer -Q 33 -f 4 -l 104 -i illumina/UCLGMS1233-
33669653/Sample241116-41186489/Sample241116_S1_L001_R1_001.fastq -o
illumina/trim.fastq
```

2- **Quality Control** – Keep all reads with >30 quality value over >90% of their sequence, discard the rest (fastq_quality_filter is from the FASTX-Toolkit, available at: http://hannonlab.cshl.edu/fastx_toolkit/)

```
fastq_quality_filter -Q 33 -q 30 -p 90 -v -i illumina/trim.fastq -o
illumina/q30_trim.fastq
```

Input: 14207534 reads.
Output: 13263866 reads.
The read counts for each library after quality filtering are in Supplementary Table 5.

3- **Demultiplexing** – Use the indices in the input text file to divide the fastq file containing all the reads into separate files for each sequencing library.

```
cat illumina/q30_trim.fastq | fastx_barcode_splitter.pl --bol --
exact --bcfile illumina/BC_161125.txt --prefix illumina/split/ --
suffix .fq
```

4- **Trim each file** – Remove indices from each file

```
fastx_trimmer -Q 33 -f 7 -l 93 -i illumina/split/1g_pre.fq -o
illumina/split/1g_pre_trim.fq
```

5- **Further trimming** – Remove fixed regions and ensure all libraries could be translated in the +1 frame. Each library was individually inspected to determine the number of residues that needed to be removed.

```
fastx_trimmer -f 46 -Q 33 -i 1g_post_trim.fq -o 1g_post_frame1.fq
```

6- **Convert from fastq to fasta** – The translation package uses a fasta input.

```
./bin/fastq_to_fasta -Q 33 -i frame/control-frame1.fq -o
frame/fasta/control.fasta
```

7- **Translate in frame 1** – transeq is from the EMBOSS package (v 6.6.0)[32]. The output is a FASTA file containing each read translated in frame +1 from nucleic acid to protein sequences.

```
transeq -sequence control.fasta -outseq control_translated.fasta
```

8- **Remove C-terminal fixed regions** – Custom perl script Cter-trim.pl was written to remove any sequence after the target in the W-loop that was not diversified. Since the length of the variable region is not constant, this could not be done by fixed-length trimming and the sequence of the fixed region had to be matched. The read counts for each library after this step are in Supplementary Table 5.

```
perl Cter-trim.pl 1g_pre.fasta 1g_pre-trim.fasta
```

9- **Count reads** – Custom perl script fasta-to-fastaCounts.pl was written to count unique reads, output is a .fasta file containing each unique sequence with the number of times it was repeated in the sequencing dataset in its header.

```
perl fasta-to-fastaCounts.pl 1g_pre-trim.fasta 1g_pre-counts.fasta
```

10- **Convert fasta counts files into CSV files** – Custom perl script fastaCounts-to-Matlab.pl was written to transform the data into a .csv format that could be used as input for the MATLAB analysis pipeline. Outputs contain the raw sequences, the same sequences with Zs added to each end for k-mer analysis and –Exc file containing all sequences with mutations that disrupted the C-terminal fixed region and prevented accurate analysis.

```
perl fastaCounts-to-Matlab.pl ../TrimTransSeqs/1g_post-counts.fasta
1g_postML.csv 1g_postML-Z.csv 1g_postML-Exc.csv
```

11- **Calculate Z-scores for post-selection enrichment** - A Z-score was calculated for each pair of pre- and post-selection libraries to compare the distributions and identify highly-enriched sequences, using a Poisson distribution to model each sequencing dataset. MATLAB script TEM1_PoissonZscores_csvOutput.m takes in the .csv files created in the previous step and a third .csv file containing the total number of reads in each library. The output of this script is a .csv file containing the sequences from the post-selection library, followed by their counts in both libraries and the calculated Z-score.

12- **Kmer-based PCA** - InDEL_analysis_Poisson.m takes the .csv output of the previous script and carries out the k-mer based PCA analysis of selection, using the separate function pc.m

13- **Hamming Distance calculations** - Custom perl script fastaCounts-HammD.pl calculates Hamming distances from an input origin sequence to all sequences of the same length from an input fasta file, producing a .csv file containing all sequences of the same length as the seed, their counts and Hamming distances to the seed.

```
perl fastaCounts-HammD.pl [Hamming distance origin sequence]
   1g_pre-counts.fasta 1g_pre-HammD.csv
```

Supplementary Note References

34. Rice, P., Longden, I. & Bleasby, A. EMBOSS: The European Molecular Biology Open Software Suite. *Trends Genet* **16**, 276-277 (2000).
